# Design, Synthesis, and Biological Evaluation of Novel Tetrahydroacridin Hybrids with Sulfur-Inserted Linkers as Potential Multitarget Agents for Alzheimer’s Disease

**DOI:** 10.3390/molecules29081782

**Published:** 2024-04-14

**Authors:** Xiuyuan Wu, Xiaotong Ze, Shuai Qin, Beiyu Zhang, Xinnan Li, Qi Gong, Haiyan Zhang, Zheying Zhu, Jinyi Xu

**Affiliations:** 1 State Key Laboratory of Natural Medicines, Department of Medicinal Chemistry, China Pharmaceutical University, 24 Tong Jia Xiang, Nanjing 210009, China; wxy15093080467@163.com (X.W.); zexiaotongx@163.com (X.Z.); qs15850672957@163.com (S.Q.); xinnanli@126.com (X.L.); 2 Therapeutics & Formulation, School of Pharmacy, The University of Nottingham, University Park Campus, Nottingham NG7 2RD, UK; beiyu.zhang@nottingham.ac.uk; 3 CAS Key Laboratory of Receptor Research, Shanghai Institute of Materia Medica, Chinese Academy of Sciences, 555 Zu Chong Zhi Road, Shanghai 201203, China; gq1021@simm.ac.cn (Q.G.); hzhang@simm.ac.cn (H.Z.)

**Keywords:** Alzheimer’s disease, tacrine, cystamine, MTDLs, inhibitors, molecular modeling

## Abstract

Alzheimer’s disease (AD) is a complex neurodegenerative disease that can lead to the loss of cognitive function. The progression of AD is regulated by multiple signaling pathways and their associated targets. Therefore, multitarget strategies theoretically have greater potential for treating AD. In this work, a series of new hybrids were designed and synthesized by the hybridization of tacrine (**4**, AChE: IC_50_ = 0.223 μM) with pyrimidone compound **5** (GSK-3*β*: IC_50_ = 3 μM) using the cysteamine or cystamine group as the connector. The biological evaluation results demonstrated that most of the compounds exhibited moderate to good inhibitory activities against acetylcholinesterase (AChE) and glycogen synthase kinase 3*β* (GSK-3*β*). The optimal compound **18a** possessed potent dual AChE/GSK-3*β* inhibition (AChE: IC_50_ = 0.047 ± 0.002 μM, GSK-3*β*: IC_50_ = 0.930 ± 0.080 μM). Further molecular docking and enzymatic kinetic studies revealed that this compound could occupy both the catalytic anionic site and the peripheral anionic site of AChE. The results also showed a lack of toxicity to SH-SY5Y neuroblastoma cells at concentrations of up to 25 μM. Collectively, this work explored the structure–activity relationships of novel tetrahydroacridin hybrids with sulfur-inserted linkers, providing a reference for the further research and development of new multitarget anti-AD drugs.

## 1. Introduction

Alzheimer’s disease (AD) is the most common form of dementia, accounting for 50–70% of cases [1,2,3]. It is characterized by impairments in language, memory, and cognition, which seriously affect patients’ quality of life [4,5,6,7,8]. The number of people with dementia worldwide is expected to reach 152.8 million by 2050, with the largest increase in low- and middle-income countries [9]. The pathogenesis of AD is still unclear. Several hypotheses have been proposed, mainly including the cholinergic injury hypothesis, tau protein hyperphosphorylation hypothesis, A*β* toxicity hypothesis, oxidative stress, metal ion disorder hypothesis, and inflammation hypothesis [10,11,12,13]. The complex pathogenesis of AD makes it difficult to develop new drugs, and many attempts have failed.

To date, several key targets regulating the generation and deterioration of AD have been discovered, such as acetylcholinesterase (AChE), monoamine oxidase B (MAO-B), glycogen synthase kinase 3*β* (GSK-3*β*), brain metal ions, phosphodiesterases (PDEs), and the *N*-methyl-D-aspartate (NMDA) receptor [14,15,16]. These targets and their related signaling pathways can influence disease progression individually or interactively [17,18,19]. The multitarget-directed ligand (MTDL) strategy is a single chemical entity that is able to target two or more AD-related targets. The MTDL strategy has been applied by several research groups, with encouraging results [20]. Researchers believe that MTDLs may be the best pharmacological option for treating AD [14]. The cholinergic injury hypothesis and tau protein hyperphosphorylation hypothesis are widely accepted by researchers, and the corresponding AChE and GSK-3*β* are two important targets in the treatment of AD. Therefore, developing dual AChE/GSK-3*β* inhibitors is a promising strategy, and many research groups have been involved in this work. [21,22,23,24,25,26]. On the one hand, the concentration of acetylcholine (ACh) in the synaptic cleft is increased to achieve cognitive improvement [27,28]. On the other hand, it can inhibit the hyperphosphorylation of tau proteins and reduce the accumulation of neurofibrillary tangles (NFTs) [29]. Therefore, it has the potential to exert better therapeutic effects than single-target drugs [30,31].

**Figure 1:**
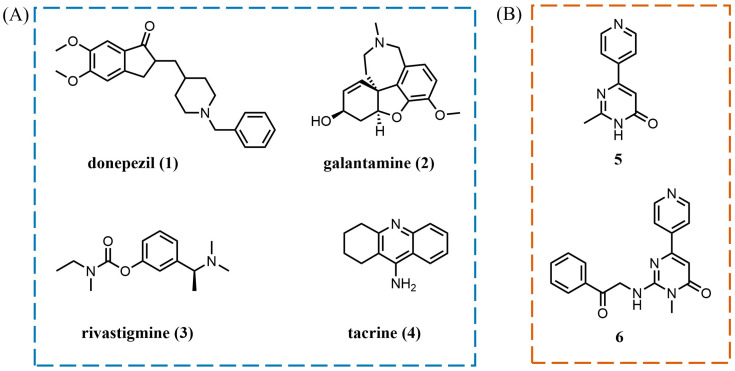
(**A**) Drugs approved by the FDA for the treatment of AD and (**B**) structures of GSK-3*β* inhibitors.

The cholinergic injury hypothesis is a classic guideline for AD, first reported by Davies and Maloney in 1976 [32]. This hypothesis suggests that the damage of basal forebrain cholinergic neurons caused by various factors, and the impairment of cholinergic neurotransmission in related cortical and hippocampal brain regions play an important role in the process of memory and cognitive impairment [33,34,35]. ACh is temporarily deposited in synaptic vesicles. After receiving nerve signals, it is released into the synaptic space and binds to cholinergic receptors, thus realizing the conduction of cholinergic nerve signals between different neurons [36,37,38]. ACh in the synaptic gap is captured by AChE after completing the signaling process and is subsequently hydrolyzed [39,40,41,42]. In pathological conditions, the catalytic activity of AChE is upregulated, resulting in a relative decrease in ACh concentration in the synaptic gap, and thus inhibits the transmission of cholinergic signals between neurons [43]. Inhibiting AChE activity to prevent ACh degradation in synapses is the most important approach in medicinal chemistry, which makes AChE an important target for regulating AD [44]. Drugs approved by the FDA for the treatment of AD are donepezil (**1**), galantamine (**2**), rivastigmine (**3**), and tacrine (**4**), all of which are AChE inhibitors (Figure 1A) [2]. They can significantly improve the cognitive function of patients but cannot prevent the progression of dementia or cure AD [45]. Tacrine (**4**) was approved in 1993 as the first AChE inhibitor for the treatment of AD and was withdrawn shortly after due to hepatotoxicity [46,47]. However, it is still being modified by multiple research groups [48] to find potent AChE inhibitors with low toxicities.

Meanwhile, GSK-3*β* is hyperactivated in the brains of AD patients [49,50,51,52]. As the main tau kinase involved in AD pathology [53], it is responsible for the hyperphosphorylation of tau proteins, an important component of NFTs [54,55,56,57], which makes it a key factor in the pathogenesis of AD [58]. Studies have shown that the cholinergic injury hypothesis is closely related to tau pathology [24,27]. GSK-3*β* can regulate the hyperphosphorylation of tau proteins, thereby affecting the distribution of choline acetyltransferase (ChAT), which further affects the formation and activity of AChE [59]. AChE activity is related to tau hyperphosphorylation, and GSK-3*β*-mediated tau hyperphosphorylation increases the protein expression of AChE [60]. AChE can disrupt cholinergic system function and interact with tau to accelerate the accumulation of NFTs. Since both AChE and GSK-3*β* are associated with the etiology and development of AD [61,62,63], an effective strategy for the treatment of AD may be to design dual AChE/GSK-3*β* inhibitors. Compound **5** was screened as a GSK-3*β* inhibitor (Figure 1B) [64]. Docking studies showed that the 2-methyl group on pyrimidone was located in the outer region of the ATP-binding pocket, and through further modification, compound **6** was obtained. It was thus identified that the pyrimidone fragment was important for the inhibitory activities [64].

## 2. Results and Discussion

### 2.1. Design

**Figure 2:**
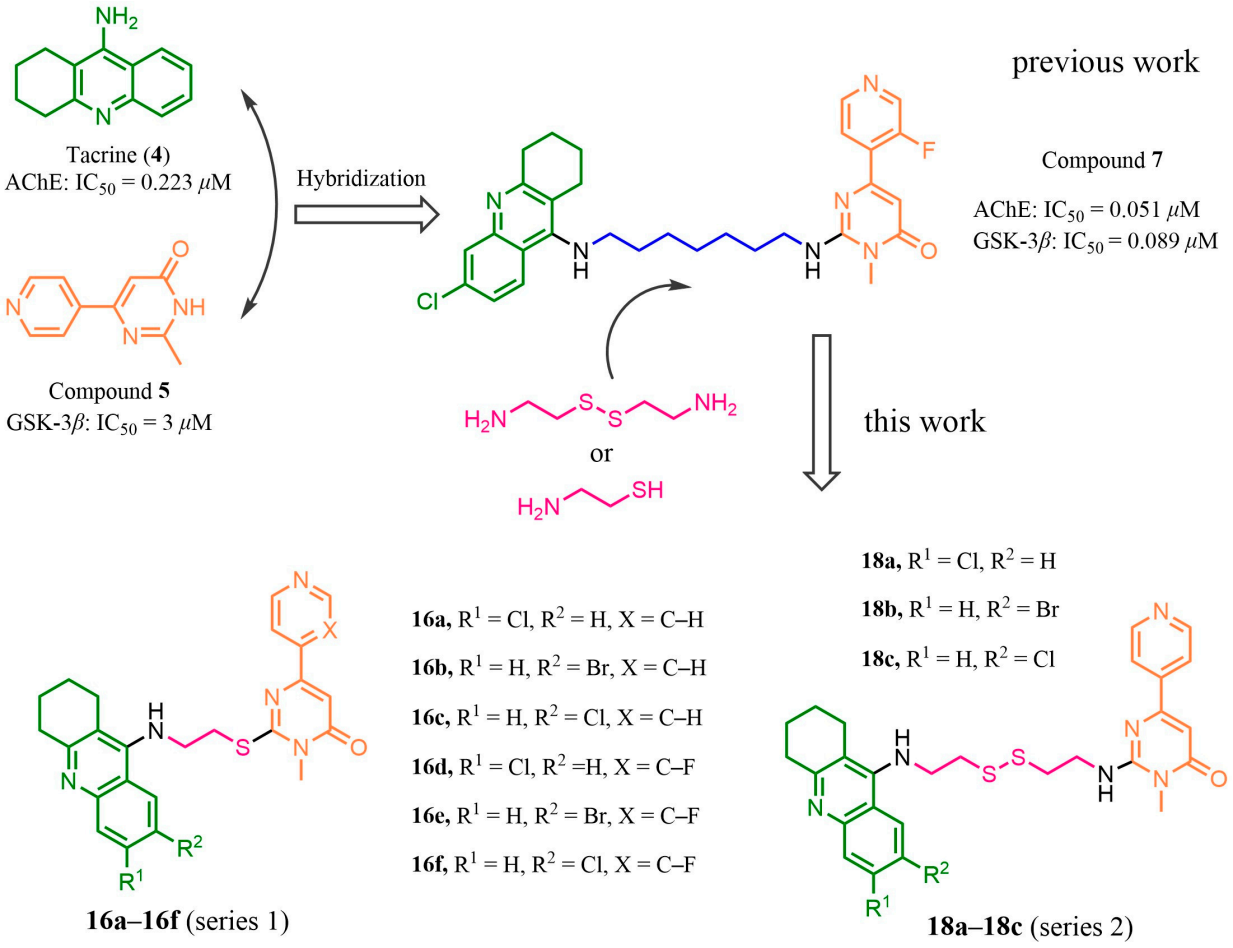
Design of novel dual AChE/GSK-3*β* inhibitors with sulfur-inserted linkers.

The active pocket on the AChE surface is narrow and deep, with the peripheral anionic site (PAS) located at the entrance and the catalytic anionic site (CAS) located at the bottom of the canyon [65,66]. Studies have shown that tacrine fragments have a high affinity for CAS [67]. Based on this, in our previous work, the active fragment with inhibitory activities against GSK-3*β* was attached to the tacrine using a suitable length of linkers, which allowed the inhibitors to occupy both the CAS and the PAS of AChE while possessing inhibitory activities against GSK-3*β*. Thus, we obtained a series of hybrids, of which compound **7** had potent activity by connecting the substituted tacrine and compound **5** with alkyl chains of different lengths [68]. It was found that inserting sulfur atoms into the linking chain can enhance the flexibility of the chain, which may make the molecules bind to proteins in a more optimized conformation [69]. Therefore, in this work, we selected the cysteamine or cystamine group to replace the heptamethylene moiety of compound **7** to further explore the structure–activity relationships (SAR); thus, a series of novel tetrahydroacridin hybrids with sulfur-inserted linkers were achieved (Figure 2), providing a reference for the further research and development of new multitarget anti-AD drugs.

### 2.2. Synthesis

**Scheme 1:**
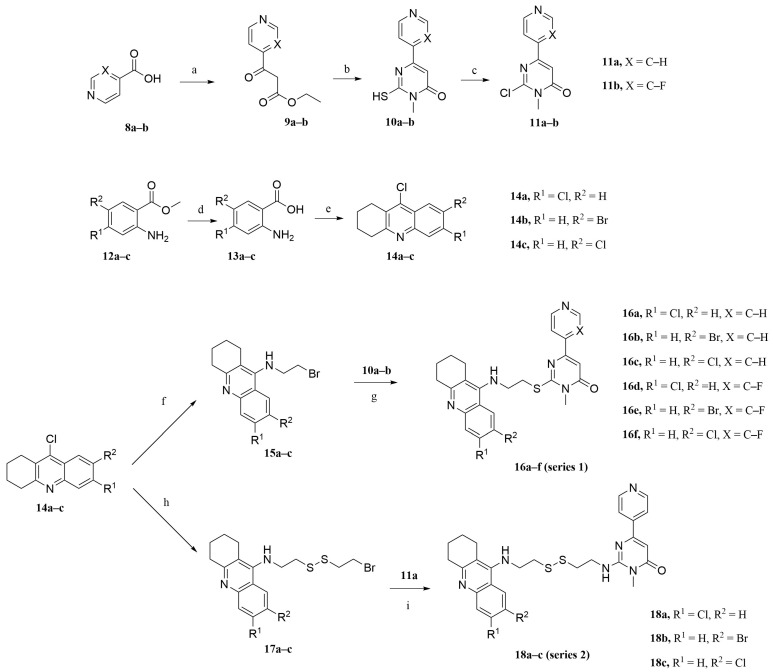
Synthesis of compounds **16a**–**16f** and **18a**–**18c**. (**a**) (i) CDI, THF, 66 °C, 1–2 h and (ii) MgCl_2_, ethyl potassium malonate, THF, 66 °C, 10 h, 45–50% yield; (**b**) EtOH, *N*-methylthiourea, DBU, 80 °C, 12 h 42–45% yield; (**c**) DCE, DMF, POCl_3_, 65 °C, 1.5 h, 48–53% yield; (**d**) (i) 2 N NaOH aqueous solution, 25 °C, 8 h and (ii) HCl, 25 °C, 20 min, 90–93% yield; (**e**) POCl_3_, cyclohexanone, 110 °C, 4 h, 75–80% yield; (**f**) *n*-pentanol, K_2_CO_3_, 2-bromoethylamine hydrobromide, 99 °C, 12 h, 50–55% yield; (**g**) CH_3_CN, K_2_CO_3_, 85 °C, 12 h, 45–50% yield; (**h**) *n*-pentanol, K_2_CO_3_, cystamine dihydrochloride, 99 °C, 12 h, 45–50% yield; and (**i**) CH_3_CN, K_2_CO_3_, 85 °C, 12 h, 43–47% yield.

The synthetic routes of compounds **16a**–**16f** and **18a**–**18c** are shown in Scheme 1. Intermediates **9a**–**b** were obtained by the reaction of commercially available agents **8a**–**b** with *N*,*N′*-carbonyldiimidazole and ethyl potassium malonate and then cycled with *N*-methylthiourea under alkaline conditions to produce **10a**–**b**. Compounds **10a**–**b** were chlorinated with POCl_3_ to obtain the key intermediates **11a**–**b**. The synthesis of the tacrine intermediate was prepared as follows: methyl anthranilates **12a**–**c** with different substituents were hydrolyzed using sodium hydroxide to produce carboxylic acids **13a**–**c**. Then, the carboxylic acids reacted with cyclohexanone under phosphorus oxychloride as a solvent to form intermediates **14a**–**c**. Intermediates **14a**–**c** reacted with 2-bromoethylamine at 137 °C to obtain intermediates **15a**–**c**, which reacted with intermediates **10a**–**b** in the presence of potassium carbonate to obtain target compounds **16a**–**f** (series 1). Intermediates **14a**–**c** reacted with cystamine dihydrochloride to produce intermediates **17a**–**c**. Intermediates **17a**–**c** reacted with **11a** to produce target compounds **18a**–**c** (series 2).

### 2.3. In Vitro Inhibitory Activities against AChE and GSK-3β

The inhibitory activities of compounds **16a**–**16f** (series 1) and **18a**–**18c** (series 2) against AChE and GSK-3*β* were evaluated in vitro. Tacrine and AR-A014418 were used as positive controls for AChE and GSK-3*β*, respectively. The IC_50_ values of all tested compounds against AChE and GSK-3*β* are summarized in Table 1.

Enzymatic assays revealed that the incorporation of tacrine fragments maintained the AChE-binding activity. Consistent with when the alkyl group was used as the connecting chain [68], the different substitutions in the benzene ring greatly affected the activity. The activity was optimal when R^1^ was substituted for Cl, and when the substituent of R^1^ was the same, the optimal substituent of R^2^ was H. The activity of compounds **18a**–**c** with the cystamine group linker was better than that of compounds **16a**–**f** with the cysteamine group linker. It was hypothesized that a longer and more flexible linker helped to ensure that the two active parts occupied the PAS and CAS sites simultaneously due to the narrow and long pocket of AChE protein. In addition, the different substitutions on the pyrimidone fragment had little effect on increasing inhibitory activities against AChE.

These hybrids showed moderate (2–40 μM) to good (0.3–2 μM) inhibitory activities against GSK-3*β*, which was consistent with our expectation. The broad and shallow active pocket of GSK-3*β* generally caused it to combine with a certain volume of molecules. The hybridization of tacrine enhanced the binding ability of inhibitors to GSK-3*β*, and replacing the alkyl link chain with the cysteamine or cystamine group retained the activity. When the cysteamine group was used as the connector, the introduction of a F atom into the pyridine ring contributed significantly to the enhanced inhibitory activities against GSK-3*β*. Similarly, the activity of the compounds with the cystamine group was more optimal than that with the cysteamine group. Optimal compound **18a** exhibited potential inhibitory activities against both AChE and GSK-3*β* and showed the best inhibition balance.

### 2.4. Kinetic Study of AChE Inhibition

**Figure 3:**
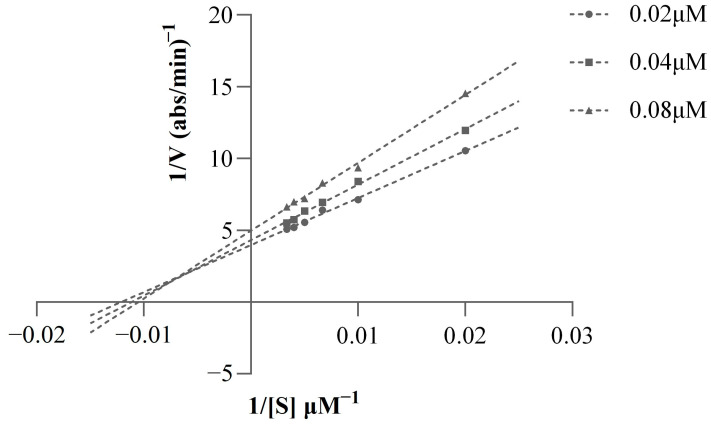
Lineweaver–Burk plot for the kinetic study of AChE inhibition by compound **18a**.

The kinetic study of the most potent compound **18a** against AChE was performed in order to determine the inhibition mode of the series of compounds. As can be observed in Figure 3, Lineweaver–Burk reciprocal plots showed that the slope and intercept also increased with the increase in inhibitor concentration, which indicated that the inhibition mode was a mixed type. Based on the inhibition mode, we suggested that compound **18a** could occupy both the CAS and the PAS of AChE.

### 2.5. Molecular Modeling Studies

**Figure 4:**
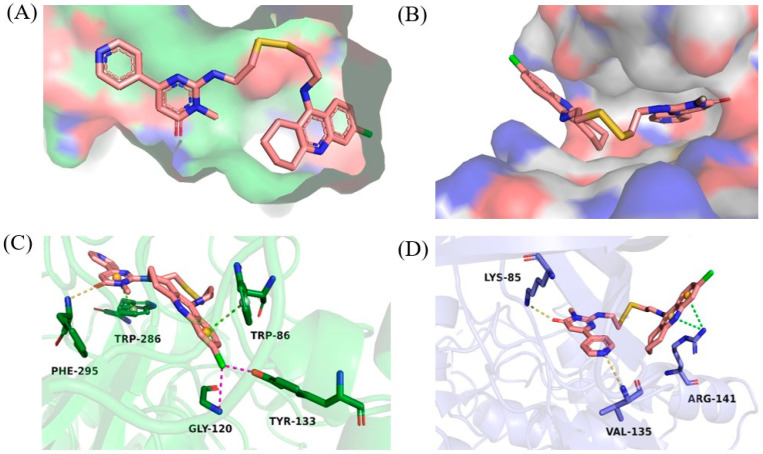
Proposed binding models of **18a** with AChE (PDB code: 4EY7) and GSK-3*β* (PDB code: 3F88). For secondary bonds, the hydrogen bond is shown as yellow dotted lines, the halogen bond is shown as purple dotted lines, and π–π stacking contacts are shown as green dotted lines. (**A**) Relative position of compound **18a** in the active pocket of AChE; (**B**) relative position of **18a** in the active pocket of GSK-3*β*; (**C**) interactions between **18a** and the amino acid residue of AChE; and (**D**) interactions between **18a** and the amino acid residue of GSK-3*β*.

Compound **18a** exhibited good inhibitory potencies against the dual targets. In order to better understand the binding patterns with the AChE and GSK-3*β* enzymes, studies of the molecular docking of compound **18a** to the proteins were performed using Schrodinger Glide, based on the reported structures of AChE (PDB ID:4EY7) and GSK-3*β* (PDB ID:3F88). The docking results after PyMOL preparation are shown in Figure 4.

We found that compound **18a** could occupy both the catalytic anion site (CAS) and the peripheral anion site (PAS) of AChE, which is consistent with the results of the kinetic study. The tacrine unit was located in the interior of the AChE gorge, and there was a π–π stacking contact between the benzene ring and Trp86, which increased the affinity for the CAS. Meanwhile, a chloride atom introduced into the tacrine unit could interact with Gly120 and Tyr133, separately, to form two halogen bonds. Compounds containing chlorine or bromine atoms were prone to forming halogen bonds because they were large enough to contain *σ* holes [70]. The pyrimidone ketone fragment was located in the exterior of the AChE gorge. The carbonyl oxygen formed a hydrogen bond interaction with Phe295, and the pyrimidine ring had a π–π stacking contact with Trp286. In addition, the linker connected the two parts at an appropriate length and conformation.

The binding mode of compound **18a** to GSK-3*β* is shown in Figure 4. The pyrimidone fragment extended to the interior of the GSK-3*β* binding groove, whereas the tacrine unit adhered to the exterior of the active pocket. Pyridine nitrogen in the pyrimidone moiety interacted with Val135 by forming a hydrogen bond, and the formation of hydrogen bonds in the hinge region was required for inhibitory activities against GSK-3*β* [71]. Meanwhile, the carbonyl oxygen formed another hydrogen bond with Lys85, which was essential for maintaining GSK-3*β* inhibition and selectivity [71]. In addition, the benzene and pyridine rings of tacrine fragments could interact with Arg141 via π–π stacking contacts, which might contribute to the better inhibition of GSK-3*β*.

### 2.6. Cytotoxicity Bioassays in HepG2 and SH-SY5Y Cell Lines

**Figure 5:**
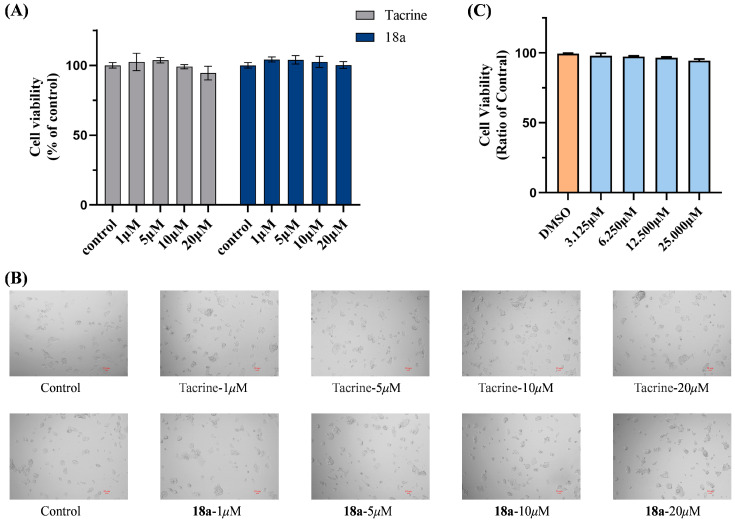
(**A**) In vitro hepatotoxicity of compound **18a** on HepG2 cell line; (**B**) microscopy images of surviving HepG2 cells under different concentrations of tacrine or compound **18a** (1 μM, 5 μM, 10 μM, and 20 μM); and (**C**) cell viability of compound **18a** on SH-SY5Y cells. All values are expressed as mean ± SEM from three independent experiments.

Tacrine was withdrawn because of its hepatotoxicity. Therefore, in this study, we focused on improving the hepatotoxicity of hybrids. The hepatotoxicity of the optimal compound **18a** was tested on HepG2 cells. As shown in Figure 5A,B, compound **18a** showed no significant hepatotoxicity at concentrations of up to 20 μM. Meanwhile, the neurotoxicity of compound **18a** on human neuroblastoma SH-SY5Y cell lines was evaluated with a 3-(4,5-dimethylthiazol-2-yl)-2,5-diphenyl-tetrazolium bromide assay. The results showed that there was no significant decrease in cell viability at the maximum test concentration of **18a** up to 25 μM (Figure 5C). The survival percentage of cells observed under the test conditions was in the range of 94.38%~98.06%, suggesting that compound **18a** showed no toxicity to SH-SY5Y neuroblastoma cells at concentrations of up to 25 μM.

### 2.7. Theoretical Prediction of the ADME Properties

It is necessary to check the potential pharmacokinetic properties of new compounds at the early stages of drug development. The success of a compound as a drug candidate depends not only on minimal toxicological effects and excellent pharmacology but also on having acceptable pharmacokinetic profiles. As shown in Table 2, the SwissADME online service (https://www.swissadme.ch/, accessed on 12 October 2023) was used to predict the drug properties of these novel hybrids. The results showed that compound **18a** tended to follow Lipinski’s rule [72], except for a molecular weight close to 500.

## 3. Materials and Methods

### 3.1. Chemistry

All reagents were chemically pure or analytically pure products and were not further purified. The ^1^H NMR and ^13^C NMR spectra were measured by a Bruker BioSpin GmbH spectrometer (Bruker Company, Karlsruhe, Germany) at 300 and 75 MHz, respectively. DMSO-*d*_6_, MeOD, or CDCl_3_ were used as solvents. TMS was used as the internal standard, the chemical shift unit was ppm, and the coupling constant unit was Hz. Proton coupling modes were described as singlet (s), broad singlet (brs), doublet (d), doublet of doublets (dd), triplet (t), and multiplet (m). The mass spectrometry (MS, HRMS) was determined by an Agilent 1100-LC-MSD-Trap/SL or an FTMS-2000 mass spectrometer (Agilent Technologies Inc., Santa Clara, CA, USA).

### 3.2. Synthetic Procedure

#### 3.2.1. Preparation of Intermediates **9a**–**9b**, **10a**–**10b**, and **11a**–**11b**

A solution of commercially available reagent **8a** (35.44 mmol, 1 eq) and *N*,*N’*-carbonyldiimidazole (38.98 mmol, 1.1 eq) in dry THF (100 mL) was stirred in a bottle for 1–2 h at 66 °C (mixture A). In another bottle, a suspension of ethyl potassium malonate (42.52 mmol, 1.2 eq) and anhydrous MgCl_2_ (42.52 mmol, 1.2 eq) in dry THF was stirred at room temperature for 1–2 h (mixture B). Then, mixture A was slowly added to mixture B, and the fresh mixture was stirred for another 10 h at 66 °C. After the completion of the reaction, 1N HCl was added to adjust the pH to 5, and the mixture was extracted with EtOAc (3 × 50 mL). The combined organic phase was washed with saturated brine, dried with anhydrous Na_2_SO_4_, and evaporated in vacuo to give the crude product, which was purified by column chromatography (petroleum ether/EtOAc = 4:1) to give a yellow solid (compound **9a**) with 45% yield. Compound **9b** was synthesized using the method for compound **9a**.

Compound **9a** (31.06 mmol, 1 eq) was dissolved in 120 mL of absolute ethanol, followed by the addition of 1, 8-diazabicyclo [5.4.0] undecarbonyl-7-ene (DBU) (77.64 mmol, 2.5 eq) and *N*-methylthiourea (34.16 mmol, 1.1 eq). The mixture was protected with nitrogen and reflux at 80 °C for 12 h, followed by TLC monitoring. After the completion of the reaction, most of the ethanol was evaporated in vacuo, and an appropriate amount of hydrochloric acid was added to precipitate the yellow solid. The resulting precipitate was filtered, washed with water, and dried under an infrared lamp to give a yellow solid (compound **10a**) without further purification (45% yield). Compound **10b** was synthesized following the method for compound **10a**.

Compound **10a** (9.12 mmol, 1 eq) was dissolved in 20 mL of a mixed solvent of 1, 2-dichloroethane and dimethylformamide with a volume ratio of 1:1, and phosphorus oxychloride (36.48 mmol, 4eq) was added under stirring. The system was heated to 65 °C and stirred for 1h. After the reaction was completed, phosphorus oxychloride was removed, dichloromethane and water were added, and the mixture was basified with saturated NaHCO_3_ solution to adjust the pH to 8. The mixture was extracted with dichloromethane (3 × 50 mL), and the incorporated organic phase was washed with saturated saline and dried over anhydrous Na_2_SO_4_. The solvent was removed in vacuo to obtain the residue, which was further purified by column chromatography (petroleum ether/EtOAc = 2:1) to give a white solid (compound **11a**) (48.5% yield). Compound **11b** was synthesized following the method for compound **11a**.

#### 3.2.2. Preparation of Intermediates **13a**–**13c** and **14a**–**14c**

The raw material **12a** (66.15 mmol) was dissolved in a sufficient amount of 2 N NaOH solution and stirred at room temperature overnight. After the reaction was completed, an appropriate amount of hydrochloric acid was added to adjust the pH to 5, and precipitation was filtered and dried to give a light yellow solid (compound **13a**) with a 91.5% yield. Compounds **13b** and **13c** were synthesized following the method for compound **13a**.

The intermediate compound **13a** (43.75 mmol, 1 eq) and cyclohexanone (48.13 mmol, 1.1 eq) were added to a 100 mL bottle. Then, phosphorus oxychloride (218.76 mmol, 5 eq) was slowly added under an ice bath, and the reaction system was stirred at 110 °C for 4h. After the reaction was completed, the reaction system was cooled to room temperature, and most of the phosphorus oxychloride was removed. Then, 2 N NaOH was added at 0 °C to regulate the pH to 9–10. The mixture was extracted with dichloromethane (3 × 50 mL), and the combined organic phases were washed with saturated brine and dried over anhydrous Na_2_SO_4_. After removing the solvents in vacuo, the left residue was purified by column chromatography (petroleum ether/EtOAc = 8:1) to give a faint yellow solid (compound **14a**) with a 75.5% yield. Compounds **14b** and **14c** were synthesized following the method for compound **14a**.

#### 3.2.3. Preparation of Intermediates **15a**–**15c**

2-bromoethylamine hydrobromide (3.57 mmol, 3 eq) and a catalytic amount of K_2_CO_3_ (5.95 mmol, 5 eq) were added to a solution of compound **14a** (1.19 mmol, 1 eq) in *n*-pentanol (5 mL), and the reaction mixture was stirred at 99 °C for 12 h. The solution was cooled to room temperature and then acidified with 3 N HCl. The mixture was extracted with water and the combined aqueous phase was basified with NaOH (pH = 8–9). Subsequently, the aqueous phase was extracted with dichloromethane (4 × 50 mL), and the combined organic layers were washed with brine, dried over anhydrous Na_2_SO_4_, and concentrated in vacuo to give a crude product, which was purified by column chromatography (dichloromethane/methanol = 50:1) to provide a yellow oil (compound **15a**) with 53% yield. Compounds **15b** and **15c** were synthesized following the method for compound **15a**.

#### 3.2.4. General Procedures for the Synthesis of Target Compounds **16a**–**16f**

Compound **10a** (0.35 mmol, 1.2 eq) and potassium carbonate (0.89 mmol, 3 eq) were added to a solution of compound **15a** (0.3 mmol, 1 eq) in acetonitrile (6 mL), and the reaction mixture was stirred at 85 °C for 12 h. The mixture was filtered to remove potassium carbonate, removed acetonitrile in vacuo, and extracted with dichloromethane (3 × 20 mL). The organic layer was washed with saturated brine and concentrated. The corresponding target compound **16a** was purified by column chromatography (dichloromethane/methanol = 20:1) with a 50% yield. Compounds **16b**, **16c**, **16d**, **16e**, and **16f** were synthesized following the method for compound **16a**.

#### 3.2.5. Preparation of Intermediates **17a**–**17c**

Cystamine dihydrochloride (3.57 mmol, 3 eq) and a catalytic amount of K_2_CO_3_ (5.95 mmol, 5 eq) were added to a solution of compound **14a** (1.19 mmol, 1 eq) in *n*-pentanol (5 mL), and the reaction mixture was stirred at 99 °C for 12 h. The solution was cooled to room temperature and then acidified with 3 N HCl. The mixture was extracted with water, and the combined aqueous phase was basified with NaOH (pH = 8–9). Subsequently, the aqueous phase was extracted with dichloromethane (4 × 50 mL), and the combined organic layers were washed with brine, dried over anhydrous Na_2_SO_4_, and concentrated in vacuo to give a crude product, which was purified by column chromatography (dichloromethane/methanol = 50:1) to provide a yellow oil (compound **17a**) with 46% yield. Compounds **17b** and **17c** were synthesized following the method for compound **17a**.

#### 3.2.6. General Procedures for the Synthesis of Target Compounds **18a**–**18c**

Compound **11a** (0.35 mmol, 1.2 eq) and potassium carbonate (0.89 mmol, 3 eq) were added to a solution of compound **17a** (0.3 mmol, 1 eq) in acetonitrile (6 mL), and the reaction mixture was stirred at 85 °C for 12 h. The mixture was filtered to remove potassium carbonate, removed acetonitrile in vacuo, and extracted with dichloromethane (3 × 20 mL). The organic layer was washed with saturated brine and dried with anhydrous Na_2_SO_4_. The crude product was purified by column chromatography (dichloromethane/methanol = 20:1) to obtain the corresponding target compound **18a** with a 43% yield. Compounds **18b** and **18c** were synthesized following the method for compound **18a**.

### 3.3. Characterization Details

#### 3.3.1. 2-((2-((6-Chloro-1,2,3,4-tetrahydroacridin-9-yl)amino)ethyl)thio)-3-methyl-6-(pyridin-4-yl)pyrimidin-4(3H)-one (**16a**)

White solid, 35% yield; ^1^H NMR (300 MHz, Chloroform-*d*) *δ* 8.69 (d, *J* = 5.1 Hz, 2H), 7.92–7.77 (m, 2H), 7.64 (d, *J* = 5.2 Hz, 2H), 7.19 (dd, *J* = 8.9, 2.2 Hz, 1H), 6.70 (s, 1H), 4.36 (s, 1H), 3.90 (q, *J* = 6.3 Hz, 2H), 3.60 (d, *J* = 6.2 Hz, 2H), 3.56 (d, *J* = 4.6 Hz, 3H), 2.99 (t, *J* = 5.9 Hz, 2H), 2.63 (t, *J* = 5.7 Hz, 2H), 1.88–1.75 (m, 4H). ^13^C NMR (75 MHz, Chloroform-*d*) *δ* 162.3, 162.2, 159.9, 156.4, 150.7, 149.4, 147.8, 143.4, 134.3, 127.8, 124.9, 123.8, 120.5, 118.8, 117.6, 106.3, 47.8, 33.9, 33.1, 30.5, 25.0, 22.8, 22.5. HRMS-ESI (*m*/*z*): (M + H)^+^ calcd for C_25_H_25_ClN_5_OS 478.1463, found 478.1479.

#### 3.3.2. 2-((2-((7-Bromo-1,2,3,4-tetrahydroacridin-9-yl)amino)ethyl)thio)-3-methyl-6-(pyridin-4-yl)pyrimidin-4(3H)-one (**16b**)

White solid, 20% yield; ^1^H NMR (300 MHz, Chloroform-*d*) *δ* 8.69 (d, *J* = 5.2 Hz, 2H), 8.07 (s, 1H), 7.74 (d, *J* = 8.9 Hz, 1H), 7.64 (d, *J* = 5.1 Hz, 2H), 7.57 (d, *J* = 9.1 Hz, 1H), 6.71 (s, 1H), 4.30 (s, 1H), 3.88 (q, *J* = 6.3 Hz, 2H), 3.61 (d, *J* = 6.1 Hz, 2H), 3.58 (s, 3H), 2.98 (t, *J* = 5.8 Hz, 2H), 2.68 (t, *J* = 5.9 Hz, 2H), 1.89–1.81 (m, 4H). ^13^C NMR (75 MHz, Chloroform-*d*) *δ* 162.3, 162.2, 160.0, 156.3, 1507, 149.4, 143.4, 134.2, 127.8, 124.9, 123.8, 120.5, 118.8, 117.6, 106.2, 47.8, 34.0, 33.1, 30.5, 25.0, 22.8, 22.6. 13C NMR (101 MHz, Chloroform-d) δ 162.3, 162.2, 159.4, 156.3, 150.6, 148.4, 145.9, 143.4, 131.8, 130.8, 124.6, 122.0, 120.5, 118.7, 117.9, 106.3, 47.5, 33.9, 33.3, 30.5, 29.7, 25.2, 22.6. HRMS-ESI (*m*/*z*): (M + H)^+^ calcd for C_25_H_25_BrN_5_OS 522.0958, found 522.0961.

#### 3.3.3. 2-((2-((7-Chloro-1,2,3,4-tetrahydroacridin-9-yl)amino)ethyl)thio)-3-methyl-6-(pyridin-4-yl)pyrimidin-4(3H)-one (**16c**)

White solid, 25% yield; ^1^H NMR (400 MHz, Chloroform-*d*) *δ* 8.71–8.63 (m, 2H), 7.87–7.77 (m, 2H), 7.66–7.59 (m, 2H), 7.17 (dd, *J* = 9.0, 2.2 Hz, 1H), 6.69 (s, 1H), 4.36 (t, *J* = 6.6 Hz, 1H), 3.88 (q, *J* = 6.4 Hz, 2H), 3.57 (t, *J* = 6.3 Hz, 2H), 3.53 (s, 3H), 2.98 (t, *J* = 6.1 Hz, 2H), 2.62 (t, *J* = 6.0 Hz, 2H), 1.82 (d, *J* = 5.0 Hz, 4H). ^13^C NMR (75 MHz, Chloroform-*d*) *δ* 162.3, 162.2, 160.0, 156.3, 150.7, 149.4, 147.9, 143.4, 134.2, 127.8, 124.9, 123.8, 120.5, 118.8, 117.6, 106.2, 47.8, 34.0, 33.1, 30.5, 25.0, 22.8, 22.6. HRMS-ESI (*m*/*z*): (M + H)^+^ calcd for C_25_H_25_ClN_5_OS 478.1463, found 478.1470.

#### 3.3.4. 2-((2-((6-Chloro-1,2,3,4-tetrahydroacridin-9-yl)amino)ethyl)thio)-6-(3-fluoropyridin-4-yl)-3-methylpyrimidin-4(3H)-one (**16d**)

White solid, 45% yield; ^1^H NMR (300 MHz, Chloroform-*d*) *δ* 8.59–8.42 (m, 2H), 8.29 (d, *J* = 9.2 Hz, 1H), 8.14 (s, 1H), 7.80 (t, *J* = 5.9 Hz, 1H), 7.47 (s, 1H), 7.14 (d, *J* = 9.0 Hz, 1H), 6.77 (s, 1H), 4.62–4.40 (m, 2H), 3.97 (t, *J* = 5.9 Hz, 2H), 3.53 (s, 3H), 3.11 (s, 2H), 2.51 (s, 2H), 1.75 (d, *J* = 11.5 Hz, 4H). ^13^C NMR (75 MHz, Chloroform-*d*) *δ* 162.1, 161.9, 159.8, 152.2, 149.5, 147.7, 146.3, 139.9, 139.6, 134.3, 127.6, 124.9, 123.9, 122.7, 118.7, 117.5, 111.1, 111.0 47.7, 33.8, 33.0, 30.5, 25.0, 22.8, 22.5. HRMS-ESI (*m*/*z*): (M + H)^+^ calcd for C_25_H_24_ClFN_5_OS 496.1369, found 496.1397.

#### 3.3.5. 2-((2-((7-Bromo-1,2,3,4-tetrahydroacridin-9-yl)amino)ethyl)thio)-6-(3-fluoropyridin-4-yl)-3-methylpyrimidin-4(3H)-one (**16e**)

White solid, 48% yield; ^1^H NMR (300 MHz, Chloroform-*d*) *δ* 8.56 (d, *J* = 3.0 Hz, 1H), 8.44 (d, *J* = 5.1 Hz, 1H), 8.06 (d, *J* = 2.2 Hz, 1H), 7.74 (d, *J* = 8.8 Hz, 1H), 7.65 (dd, *J* = 6.6, 5.0 Hz, 1H), 7.57 (dd, *J* = 9.1, 2.1 Hz, 1H), 6.85 (s, 1H), 3.86 (q, *J* = 6.4 Hz, 2H), 3.57 (d, *J* = 2.4 Hz, 3H), 3.00 (d, *J* = 5.6 Hz, 2H), 2.74–2.63 (m, 2H), 1.84 (s, 6H). ^13^C NMR (100 MHz, Chloroform-*d*) *δ* 162.0, 161.9, 159.2, 152.1, 148.5, 146.2, 146.2, 139.9, 139.6, 130.6, 129.9, 129.3, 122.6, 121.4, 121.3, 118.6, 111.1, 111.0, 47.4, 33.9, 33.2, 30.5, 25.2, 22.8, 22.6. HRMS-ESI (*m*/*z*): (M + H)^+^ calcd for C_25_H_24_BrFN_5_OS 540.0863, found 540.0873.

#### 3.3.6. 2-((2-((7-Chloro-1,2,3,4-tetrahydroacridin-9-yl)amino)ethyl)thio)-6-(3-fluoropyridin-4-yl)-3-methylpyrimidin-4(3H)-one (**16f**)

White solid, 39% yield; ^1^H NMR (300 MHz, Chloroform-*d*) *δ* 8.55 (d, *J* = 3.1 Hz, 1H), 8.43 (d, *J* = 5.0 Hz, 1H), 7.87 (d, *J* = 2.3 Hz, 1H), 7.80 (d, *J* = 8.9 Hz, 1H), 7.63 (dd, *J* = 6.6, 5.0 Hz, 1H), 7.44 (dd, *J* = 9.0, 2.3 Hz, 1H), 6.83 (s, 1H), 4.29 (t, *J* = 6.9 Hz, 1H), 3.85 (q, *J* = 6.4 Hz, 2H), 3.56 (s, 3H), 3.53 (d, *J* = 6.1 Hz, 2H), 3.04–2.93 (m, 2H), 2.67 (t, *J* = 5.8 Hz, 2H), 1.85 (q, *J* = 4.2, 3.2 Hz, 4H). ^13^C NMR (100 MHz, Chloroform-*d*) *δ* 162.0, 161.9, 159.1, 152.1, 148.5, 146.2, 146.2, 145.6, 139.9, 139.6, 130.6, 129.9, 129.3, 122.6, 121.3, 118.6, 111.1, 111.0, 47.4, 33.9, 33.1, 30.5, 25.2, 22.8, 22.6. HRMS-ESI (*m*/*z*): (M + H)^+^ calcd for C_25_H_24_ClFN_5_OS 496.1369, found 496.1363.

#### 3.3.7. 2-((2-((2-((6-Chloro-1,2,3,4-tetrahydroacridin-9-yl)amino)ethyl)disulfanyl)ethyl)amino)-3-methyl-6-(pyridin-4-yl)pyrimidin-4(3H)-one (**18a**)

White solid, 10% yield; ^1^H NMR (400 MHz, Methanol-*d*_4_) *δ* 8.55–8.49 (m, 2H), 8.07 (d, *J* = 2.3 Hz, 1H), 7.90–7.84 (m, 2H), 7.69 (d, *J* = 9.0 Hz, 1H), 7.52 (dd, *J* = 9.0, 2.2 Hz, 1H), 6.34 (s, 1H), 3.88–3.76 (m, 4H), 3.39 (s, 3H), 3.03–2.96 (m, 4H), 2.94 (t, *J* = 6.1 Hz, 2H), 2.68 (t, *J* = 5.8 Hz, 2H), 1.91–1.81 (m, 4H). ^13^C NMR (100 MHz, Methanol-*d*_4_) *δ* 164.6, 163.8, 157.8, 154.2, 149.1, 145.2, 145.1, 144.0, 129.4, 129.20, 127.8, 122.2, 121.1, 116.8, 116.2, 98.5, 41.2, 38.4, 37.0, 32.3, 26.6, 26.4, 24.8, 22.4, 22.0. HRMS-ESI (*m*/*z*): (M + H)^+^ calcd for C_27_H_30_ClN_6_OS_2_ 553.1606, found 553.1619.

#### 3.3.8. 2-((2-((2-((7-Bromo-1,2,3,4-tetrahydroacridin-9-yl)amino)ethyl)disulfanyl)ethyl)amino)-3-methyl-6-(pyridin-4-yl)pyrimidin-4(3H)-one (**18b**)

White solid, 19% yield; ^1^H NMR (500 MHz, DMSO-*d*_6_) *δ* 8.61 (d, *J* = 5.2 Hz, 2H), 8.45 (s, 1H), 7.90 (d, *J* = 5.4 Hz, 2H), 7.77 (s, 2H), 7.65 (t, *J* = 5.7 Hz, 1H), 6.40 (s, 1H), 3.87 (s, 2H), 3.70 (t, *J* = 6.7 Hz, 2H), 3.29 (s, 3H), 3.16 (s, 1H), 3.06 (t, *J* = 7.0 Hz, 2H), 2.99 (t, *J* = 7.1 Hz, 2H), 2.92 (d, *J* = 6.6 Hz, 2H), 2.68 (t, *J* = 6.2 Hz, 2H), 1.76 (dq, *J* = 17.7, 6.9, 5.0 Hz, 4H). ^13^C NMR (125 MHz, DMSO-*d*_6_) *δ* 162.8, 161.8, 157.1, 154.6, 150.5, 148.8, 146.6 144.6, 130.4, 129.1, 125.0, 123.0, 121.0, 120.0, 116.0, 98.4, 47.0, 41.4, 38.3, 37.2, 29.4, 27.9, 25.6, 22.6, 21.9. HRMS-ESI (*m*/*z*): (M + H)^+^ calcd for C_27_H_30_BrN_6_OS_2_ 597.1100, found 597.1105.

#### 3.3.9. 2-((2-((2-((7-Chloro-1,2,3,4-tetrahydroacridin-9-yl)amino)ethyl)disulfanyl)ethyl)amino)-3-methyl-6-(pyridin-4-yl)pyrimidin-4(3H)-one (**18c**)

White solid, 15% yield; ^1^H NMR (400 MHz, Methanol-*d*_4_) *δ* 8.55–8.48 (m, 2H), 8.07 (d, *J* = 2.3 Hz, 1H), 7.90–7.85 (m, 2H), 7.69 (d, *J* = 9.0 Hz, 1H), 7.52 (dd, *J* = 9.0, 2.3 Hz, 1H), 6.34 (s, 1H), 3.88–3.76 (m, 4H), 3.39 (s, 3H), 3.02–2.96 (m, 4H), 2.94 (t, *J* = 6.0 Hz, 2H), 2.68 (t, *J* = 5.8 Hz, 2H), 1.91–1.82 (m, 4H). ^13^C NMR (100 MHz, Chloroform-*d*) *δ* 163.3, 159.4, 157.8, 153.8, 150.2, 147.6, 147.3, 144.6, 134.5, 127.3, 124.8, 124.4, 120.8, 118.5, 117.1, 99.7, 46.8, 41.1, 39.0, 37.0, 33.8, 27.3, 24.8, 22.8, 22.5. HRMS-ESI (*m*/*z*): (M + H)^+^ calcd for C_27_H_30_ClN_6_OS_2_ 553.1606, found 553.1623.

### 3.4. AChE Inhibition

The inhibitory activities of the compounds on AChE were determined by an Ellman assay. First, 20 μL of phosphate buffer was added to a 96-well plate. Then, compounds of different concentrations (20 μL), 100μL of 1mM DTNB, and 40 μL of AChE solution (2 U/mL) were added successively. The plate was incubated at 37 °C for 15 min. Briefly, 20 μL of acetylthiocholine iodide (1 mM) was added to the mixed solution. The plate was incubated at 25 °C for 20 min. The reaction was terminated by adding 3% SDS to each well. A microplate reader (FC/K3, Thermo, Waltham, MA, USA) was used to record the absorbance at 405 nm. The inhibition of each compound was calculated using the formula (1 − Ai/Ac) × 100, where Ai and Ac represent the absorbance of the reaction mixture in the presence and absence of inhibitors, respectively. The IC_50_ values of all target compounds were calculated by using Graph Pad Prism 9.5.

### 3.5. GSK-3β Inhibition

Add ATP and substrate polypeptide to the reaction template for GSK-3*β* under the most suitable enzyme reaction system and conditions, let the enzyme react with ATP and substrate polypeptide, and use luciferin kinase detection technology to detect enzyme activity. All the compounds to be tested were prepared by adding DMSO to a solution with a concentration of 10^−2^ mol/L and diluted with PBS to the required concentration when used; the purchased GSK-3*β* was diluted from 1600 ng/μL to 2 ng/μL, ready for use. Add the sample to be tested to the system containing the substrate polypeptide and ATP, add an appropriate amount of enzyme to other wells except the blank control well, react in a water bath at 30 °C for 30 min, add stop solution to each well to terminate the reaction, shake under dark conditions, use a microplate reader to measure the fluorescence intensity, and calculate the inhibition rate and IC_50_.

### 3.6. Kinetic Study of AChE Inhibition

In 96-well plates, 20μL of PBS, compound **18a** (20 μL) with three final concentrations (0.02 μM, 0.04 μM, and 0.08 μM), 20 μL of acetylthiocholine iodide (50–300 μM), and 40 μL of AChE (2 U/mL) were added successively. After incubation at 37 °C for 15 min, 100 μL of DTNB (1 mM) was added. A microplate reader (Thermo, Waltham, MA, USA) was used to record the absorbance at 405 nm. Lineweaver–Burk reciprocal plots were created by graphing 1/V vs. 1/[S] by using GraphPad Prism 9.5.

### 3.7. Cell Lines and Cell Culture

HepG2 cells were cultured in DMEM supplemented with 10% FBS and 100 U/mL of penicillin-streptomycin (Thermo, Waltham, MA, USA) at 37 °C in a humidified atmosphere containing 5% CO_2_.

SH-SY5Y cells were cultured in Dulbecco’s modified Eagle medium (DMEM) with F12 (KeyGEN BioTECH, Nanjing, China), supplemented with 10% (*v*/*v*) fetal bovine serum (FBS) (Sen Bei Jia) and antibiotics (penicillin streptomycin) (KeyGEN BioTECH, Nanjing, China). Cells were kept at 37 °C and 5% CO_2_ in a humidified atmosphere in a cell culture incubator and passaged twice a week.

### 3.8. Cytotoxicity Bioassays in HepG2 and SH-SY5Y Cell Lines

HepG2 cells were seeded in 96-well plates at a density of 2 × 10^4^ cells per well. After 24 h, different concentrations of tacrine or compound **18a** (1 μM, 5 μM, 10 μM, and 20 μM) and DMSO were added to the wells. MTT assay was performed, and the survival percentage of cells was calculated.

The cytotoxicity assay on SH-SY5Y cells was the same as in our previous studies. Cells were seeded in a 96-well plate, and the medium was removed after 24 h. Compound **18a** (3.125 μM, 6.25 μM, 12.5 μM, and 25 μM) was added to each well. After treatment for 24 h, 10 μL of 3-(4,5-dimethyl-2-thiazolyl)-2,5-diphenyl-2-*H*-tetrazolium bromide was added to each well. After incubation at 37 °C for 4 h, 100 μL of DMSO was added, and the absorbance of the mixture was determined at 590 nm using a microplate reader (FC/K3, Thermo, Waltham, MA, USA). We used the formula Ae/Ab 100% to calculate the survival rate, where Ae and Ab represent the absorbance in the presence and absence of the tested drugs. Each experiment was carried out three times in total.

### 3.9. Molecular Docking

Protein crystal structures were downloaded from PDB. The Preparation Wizard (Schrodinger) module was used to prepare the protein, the Grid module was used to generate the docking box, and the LigPrep module was used to prepare the ligand. The ligands were docked into the active pockets of AChE (PDB ID: 4EY7) and GSK-3*β* (PDB ID:3F88) using ultra-precision (XP) mode. The images were generated by using PyMOL.

## 4. Conclusions

A series of novel potent tetrahydroacridin hybrids with sulfur-inserted linkers were further synthesized and evaluated as potential multitarget agents for AD based on our previous work to explore the SAR. Among the target compounds, the optimal compound **18a** was found to be the most potent hybrid. Furthermore, compound **18a** showed a mixed-type inhibition activity on AChE. The docking study showed that **18a** was bound to both the CAS and the PAS of AChE. The results also showed a lack of toxicity to SH-SY5Y neuroblastoma cells at concentrations of up to 25 μM. We concluded that the introduction of sulfur-inserted linkers could help ensure the inhibitory activities in vitro, which represented a starting point for subsequent studies, also providing a reference for the further development of new multitarget anti-AD drugs. However, because compound **18a** had poor BBB permeability defect, and its hepatotoxicity has not achieved the best possible result. Thus, further modification of this series of compounds is still ongoing in our laboratory. The optimization work will focus on improving the membrane permeability and hepatotoxicity, and further hepatotoxicity assay will be performed in mice models to better understand the side effects and efficacy of new hybrids in ameliorating Alzheimer’s disease.

## Figures and Tables

**Table 1:** Structures of compounds **16a**–**16f** and **18a**–**18c** and their inhibitory activities against AChE and GSK-3*β*.

Compound	R^1^	R^2^	X	IC_50_ (μM) ± SEM ^a^	
				AChE ^b^	GSK-3*β* ^c^
16a	Cl	H	C-H	2.30 ± 0.14	15.10 ± 0.90
16b	H	Br	C-H	20.00 ± 1.50	38.50 ± 0.70
16c	H	Cl	C-H	10.30 ± 0.70	2.28 ± 0.07
16d	Cl	H	C-F	1.89 ± 0.21	1.69 ± 0.09
16e	H	Br	C-F	15.30 ± 0.90	0.94 ± 0.02
16f	H	Cl	C-F	16.50 ± 0.80	2.44 ± 0.06
18a	Cl	H		0.047 ± 0.002	0.93 ± 0.08
18b	H	Br		2.13 ± 0.11	0.37 ± 0.02
18c	H	Cl		2.24 ± 0.13	0.42 ± 0.03
Tacrine				0.229 ± 0.01	
AR-A014418 ^d^					0.222 ± 0.005

Note: ^a^ Data are expressed as the mean of three independent determinations; ^b^ the zymogen of AChE was from an electric eel; ^c^ recombinant human GSK-3*β* was used; and ^d^ a typical GSK-3*β* inhibitor was used as the positive control.

**Table 2:** ADME properties of compounds **16a**–**16f** and **18a**–**18c**.

Compound	MW ^a^	logP ^a^	tPSA(Å^2^) ^a^	HBD ^a^	HBA ^a^	Rotor ^a^	Vio ^a^
**16a**	478.01	4.48	98.00	1	4	6	0
**16b**	522.46	4.59	98.00	1	4	6	1
**16c**	478.01	4.49	98.00	1	4	6	0
**16d**	496.00	4.80	98.00	1	5	6	0
**16e**	540.45	4.82	98.00	1	5	6	1
**16f**	496.00	4.73	98.00	1	5	6	0
**18a**	553.14	4.60	135.33	2	4	10	1

Note: ^a^ MW (molecular weight), Log P (log octanol/water partition coefficient), tPSA (topological polar surface area), HBD (number of H-bond donors), HBA (number of H-bond acceptors), Rotor (number of rotatable bonds), and Vio (violation).

## Data Availability

The data used in this study are available within the article and in Supplementary Materials.

## Supplementary Materials

The following supporting information can be downloaded at: https://www.mdpi.com/article/10.3390/molecules29081782/s1, The supporting information includes characterization data (NMR spectra and HRMS spectra) and is available on the publisher’s website along with the published article.
