# Supplementary material for: Design, Synthesis, and Biological Evaluation of Novel Tetrahydroacridin Hybrids with Sulfur-Inserted Linkers as Potential Multitarget Agents for Alzheimer’s Disease
Source: Molecules. 2024 Apr 14;29(8):1782. doi: 10.3390/molecules29081782 (PMC11051924; doi:10.3390/molecules29081782)
Supplement: Supplementary file 1 [file molecules-29-01782-s001.zip › Supporting Information.pdf]

Supporting Information

Design, Synthesis and Biological Evaluation of Novel Tetrahydroacridin Hybrids with Sulfur-inserted Linkers as Potential Multitarget Agents for Alzheimer's Disease

Xiuyuan Wu[1,†], Xiaotong Ze[1,†], Shuai Qin[1], Beiyu Zhang[3], Xinnan Li[1], Qi Gong[2], Haiyan Zhang[2,*], Zheying Zhu[3,*]and Jinyi Xu[1,*]

[1] State Key Laboratory of Natural Medicines and Department of Medicinal Chemistry, China Pharmaceutical University, 24 Tong Jia Xiang, Nanjing 210009, P. R. China.

[2] CAS Key Laboratory of Receptor Research, Shanghai Institute of Materia Medica, Chinese Academy of Sciences, 555 Zu Chong Zhi Road, Shanghai 201203, China.

[3] Therapeutics & Formulation, School of Pharmacy, The University of Nottingham, University Park Campus, Nottingham NG7 2RD, UK.

* E-mails: jinyixu@china.com; hzhang@simm.ac.cn; Zheying.Zhu@nottingham.ac.uk

[†] *These authors contributed equally to this work.*

Table of contents

NMR and HRMS of compound **16a** S2

NMR and HRMS of compound **16b** S4

NMR and HRMS of compound **16c** S6

NMR and HRMS of compound **16d** S8

NMR and HRMS of compound **16e** S10

NMR and HRMS of compound **16f** S12

NMR and HRMS of compound **18a** S14

NMR and HRMS of compound **18b** S16

NMR and HRMS of compound **18c** S18

NMR and HRMS of compound **16a**

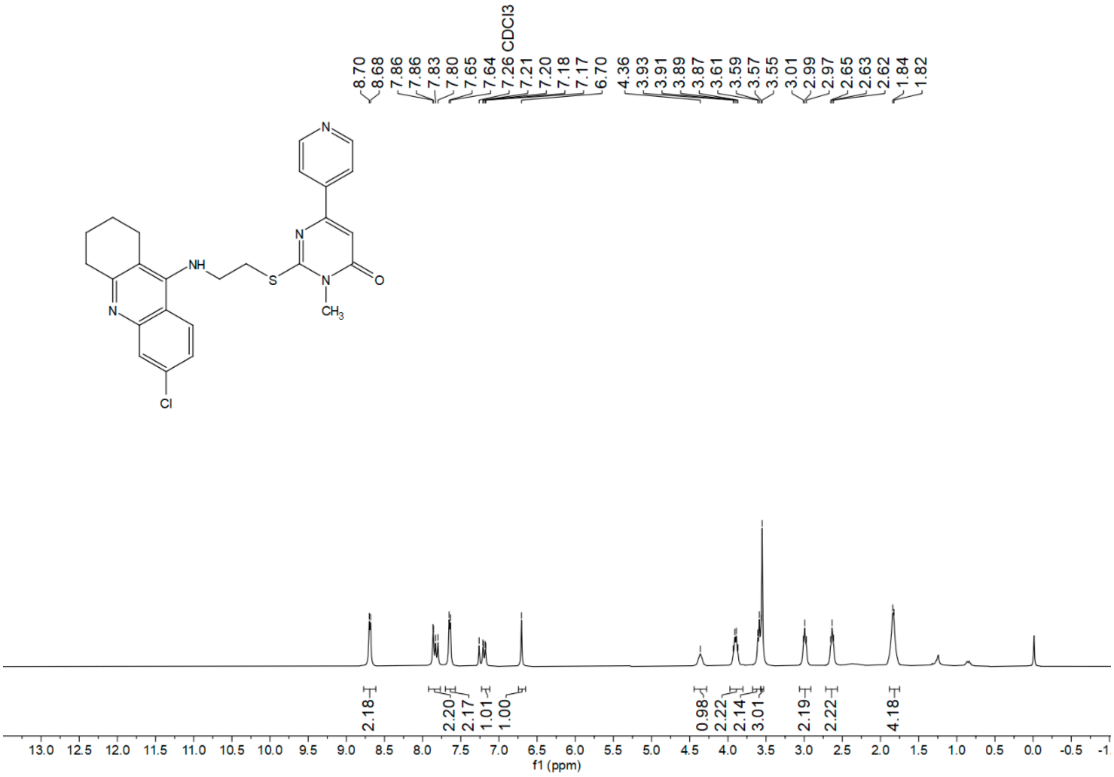

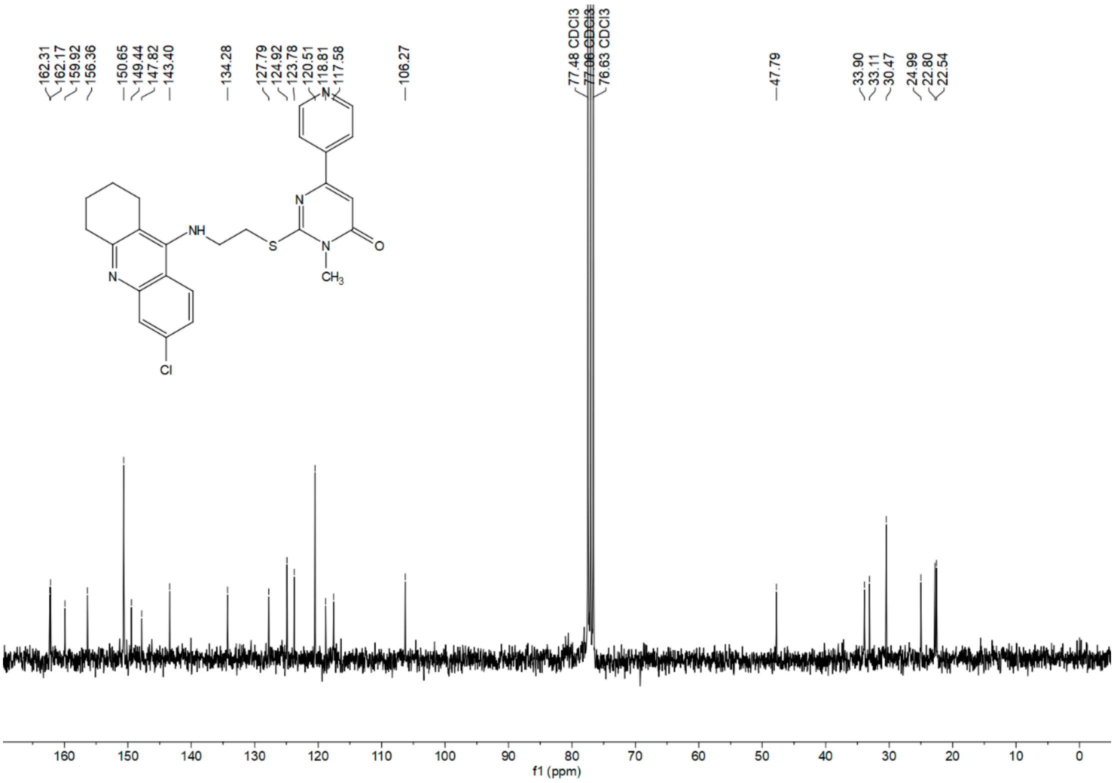

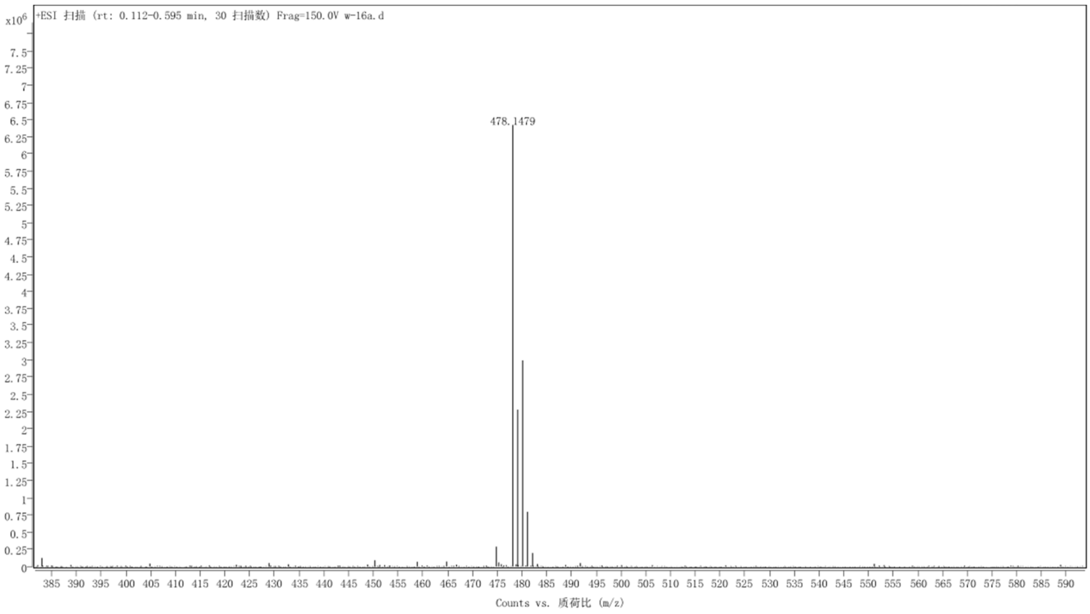

x10^6
+ESI 扫描 (rt: 0.112-0.595 min, 30 扫描数) Frag=150.0V w-16a.d
478.1479
Counts vs. 质荷比 (m/z)

NMR and HRMS of compound **16b**

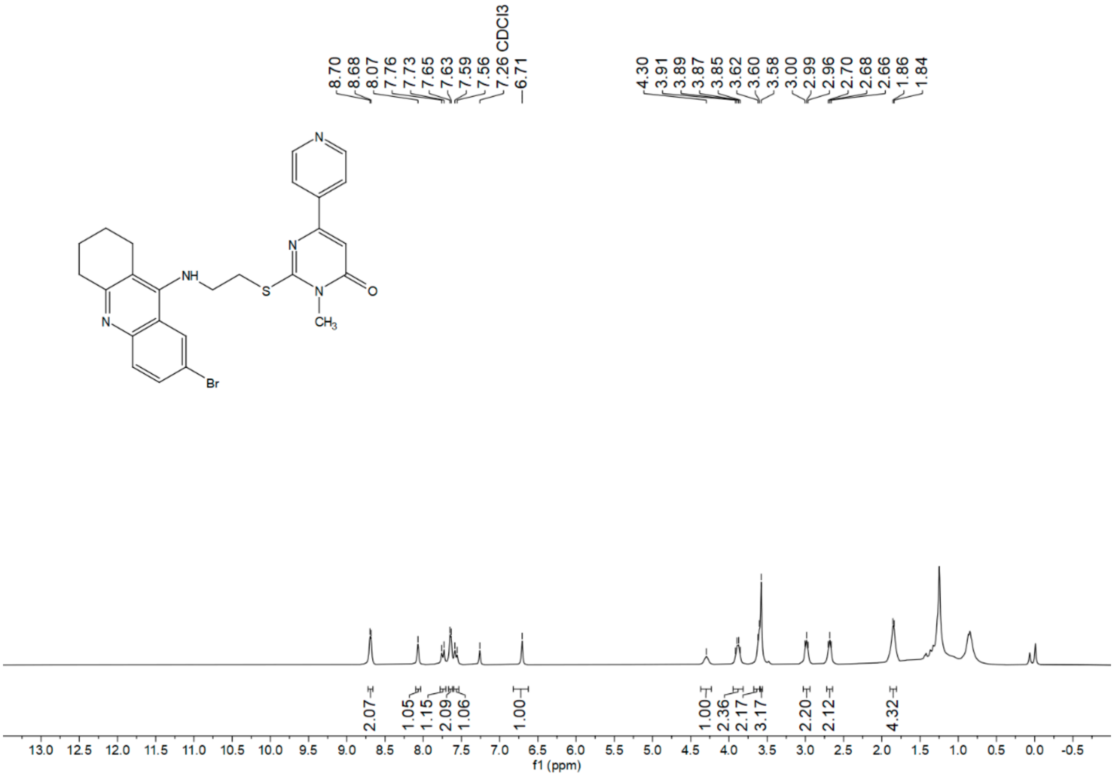

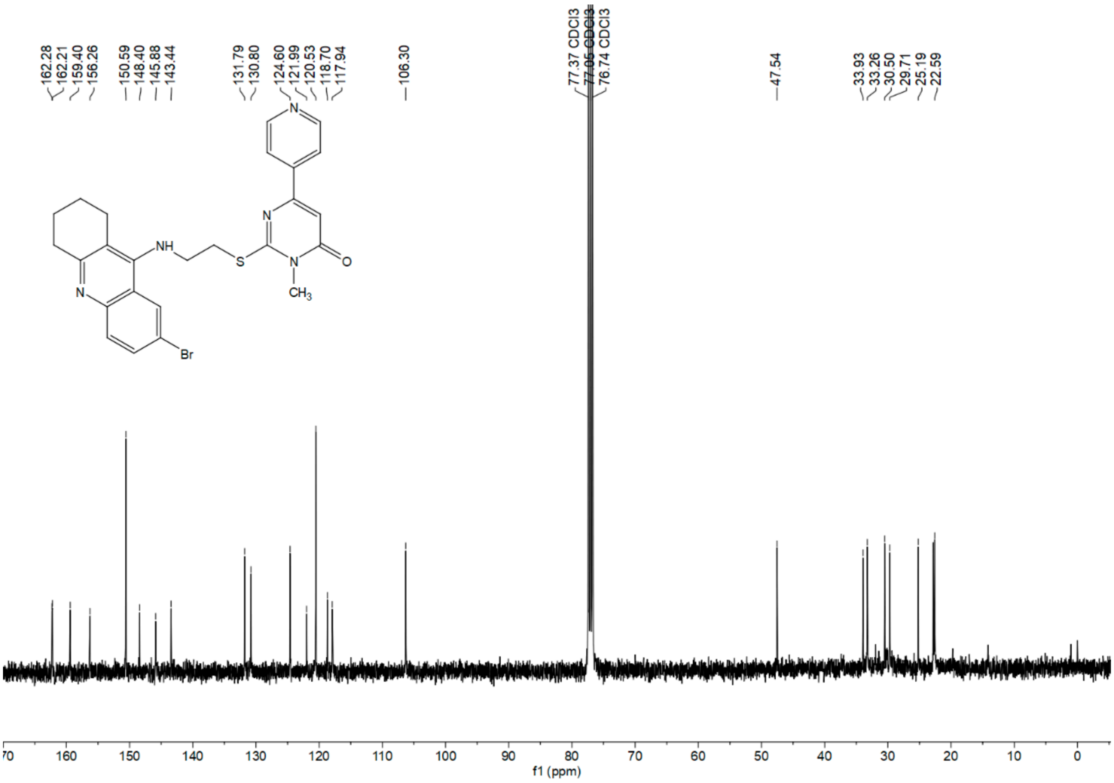

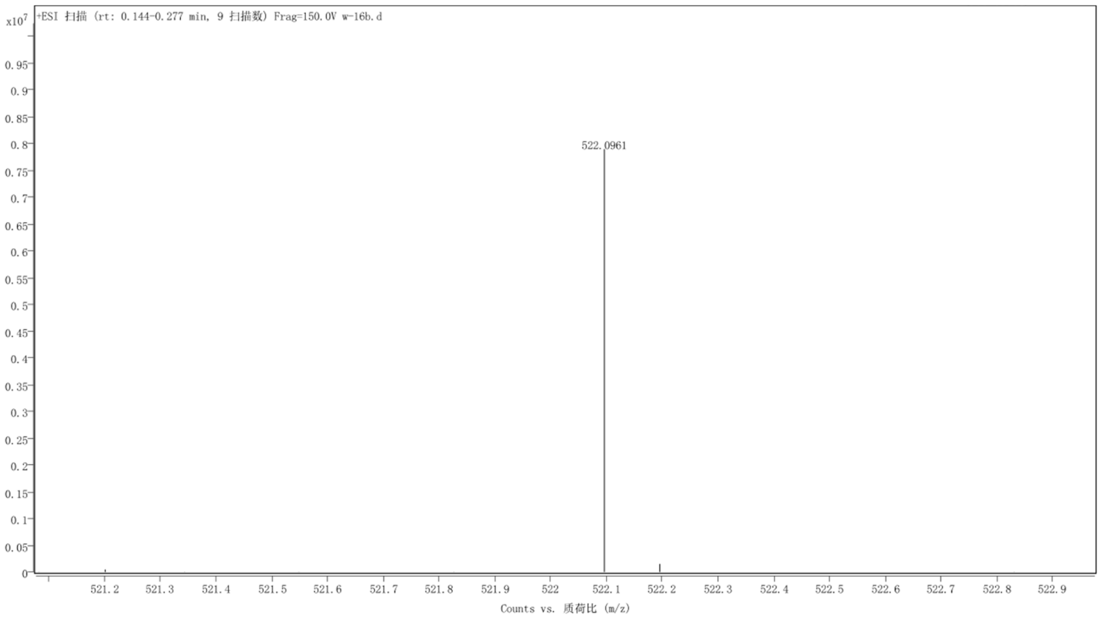
x10^7
+ESI 扫描 (rt: 0.144-0.277 min, 9 扫描数) Frag=150.0V w-16b.d
522.0961
Counts vs. 质荷比 (m/z)

NMR and HRMS of compound **16c**

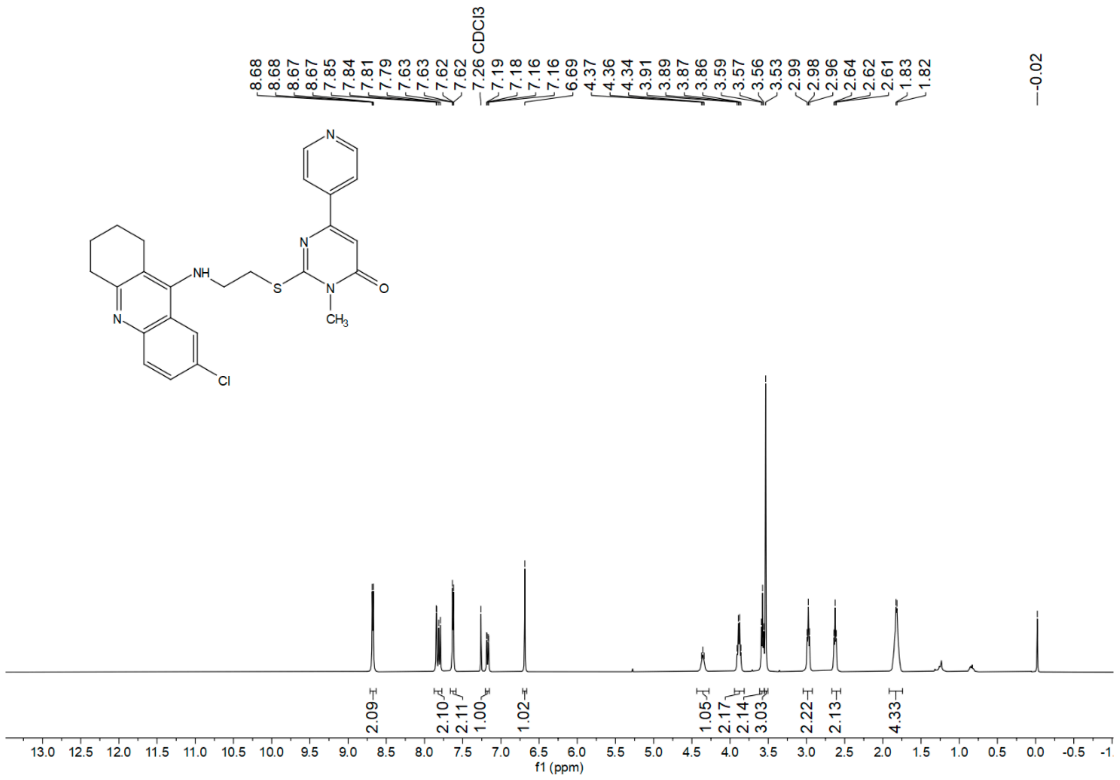

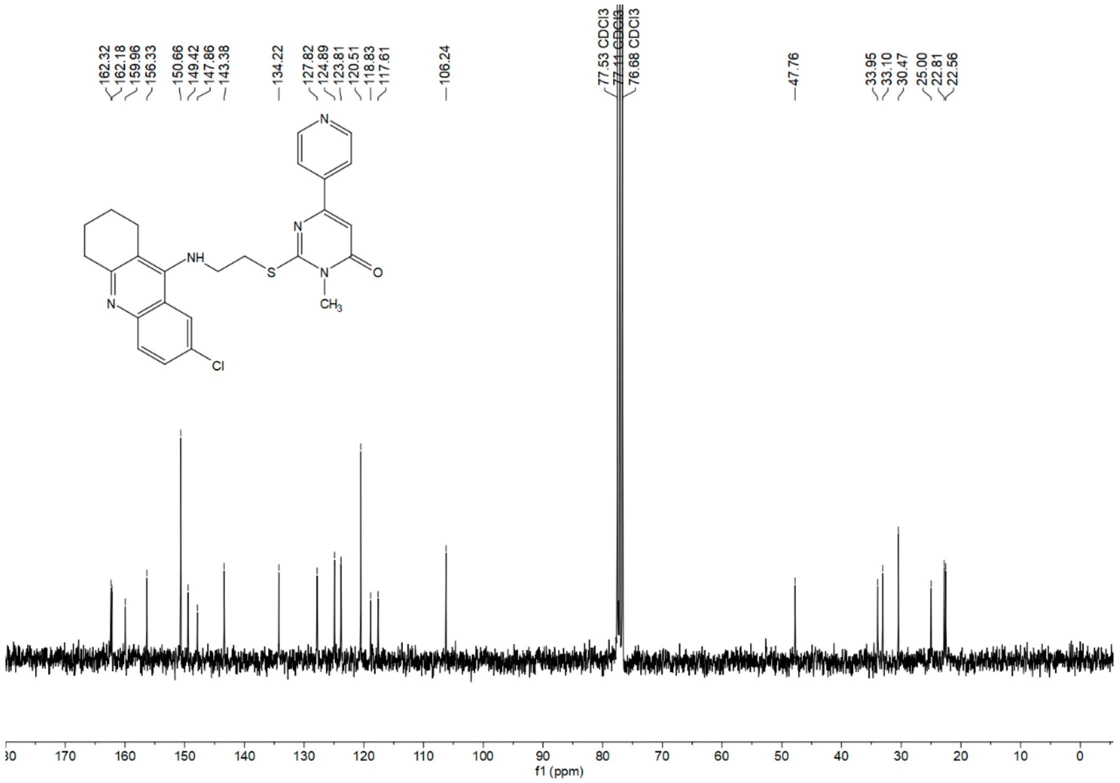

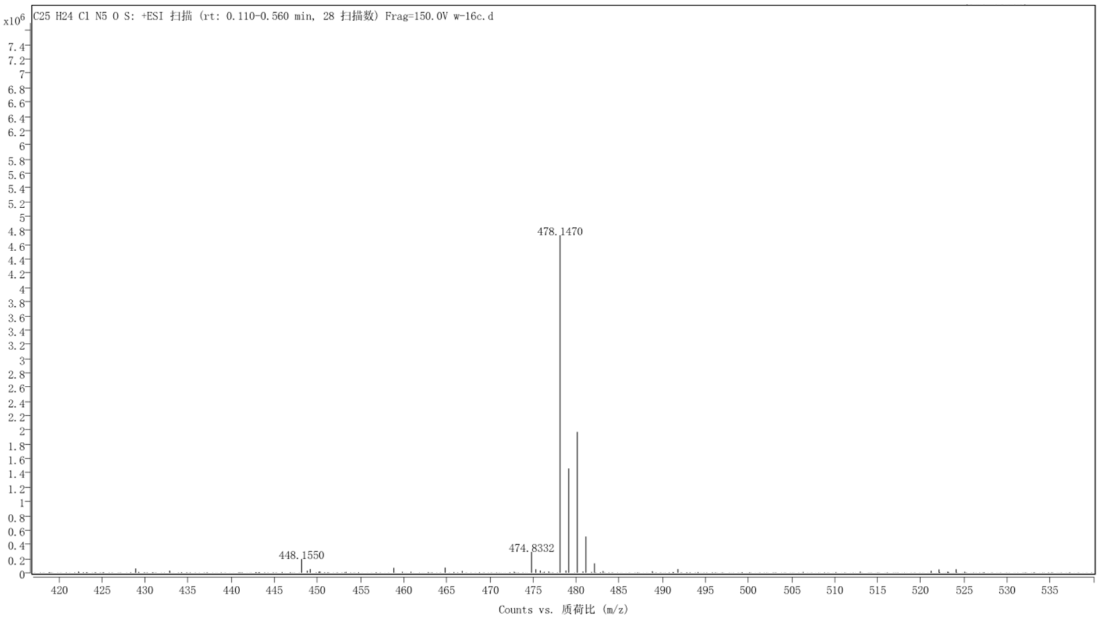

x10^6
C25 H24 Cl N5 O S: +ESI 扫描 (rt: 0.110-0.560 min, 28 扫描数) Frag=150.0V w-16c.d
478.1470
474.8332
448.1550
Counts vs. 质荷比 (m/z)

NMR and HRMS of compound **16d**

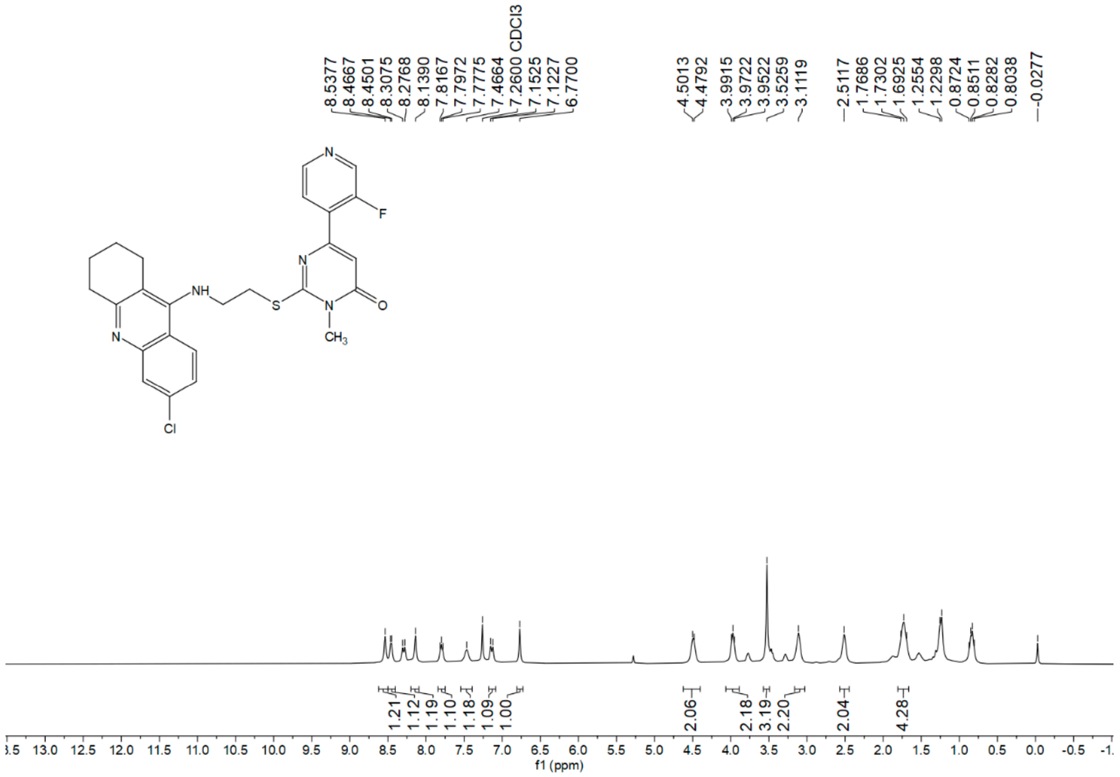

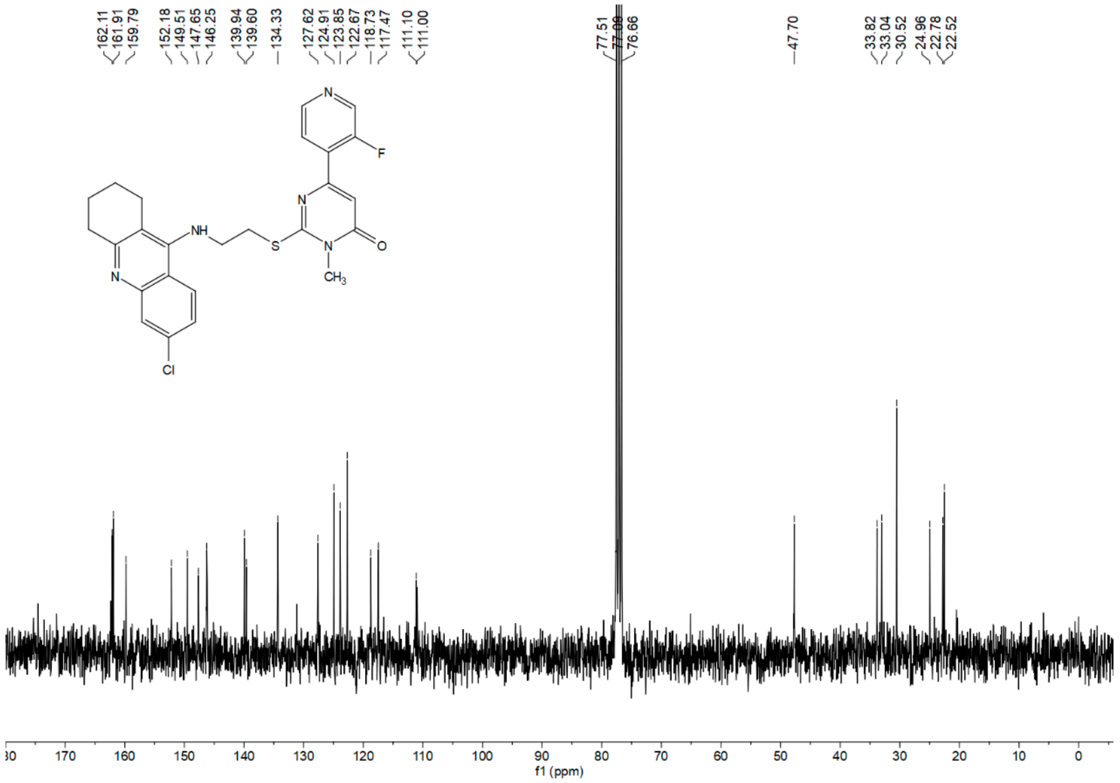

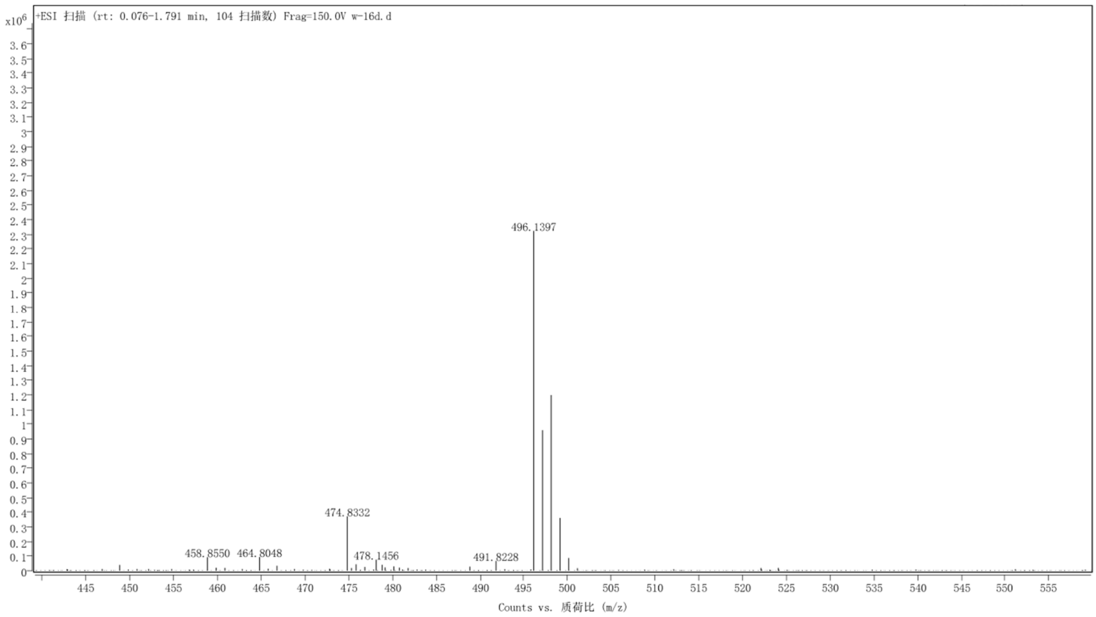

x10^6
+ESI 扫描 (rt: 0.076-1.791 min, 104 扫描数) Frag=150.0V w-16d.d
496.1397
474.8332
458.8550
464.8048
478.1456
491.8228
Counts vs. 质荷比 (m/z)

NMR and HRMS of compound **16e**

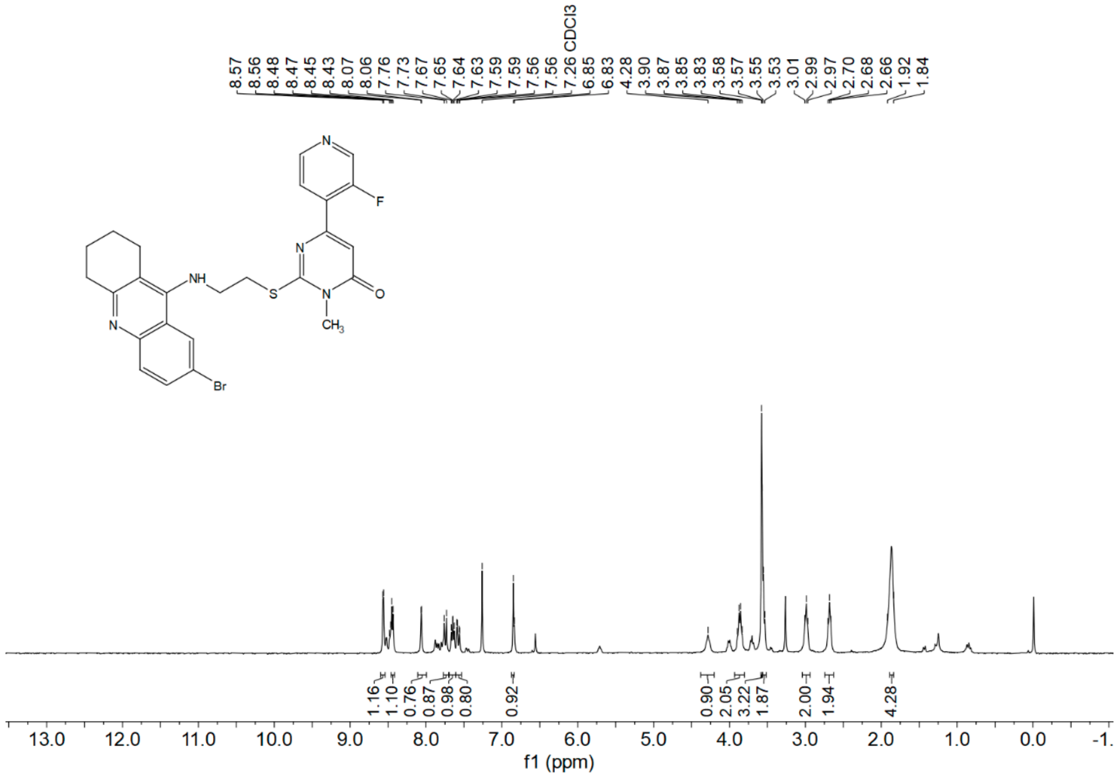

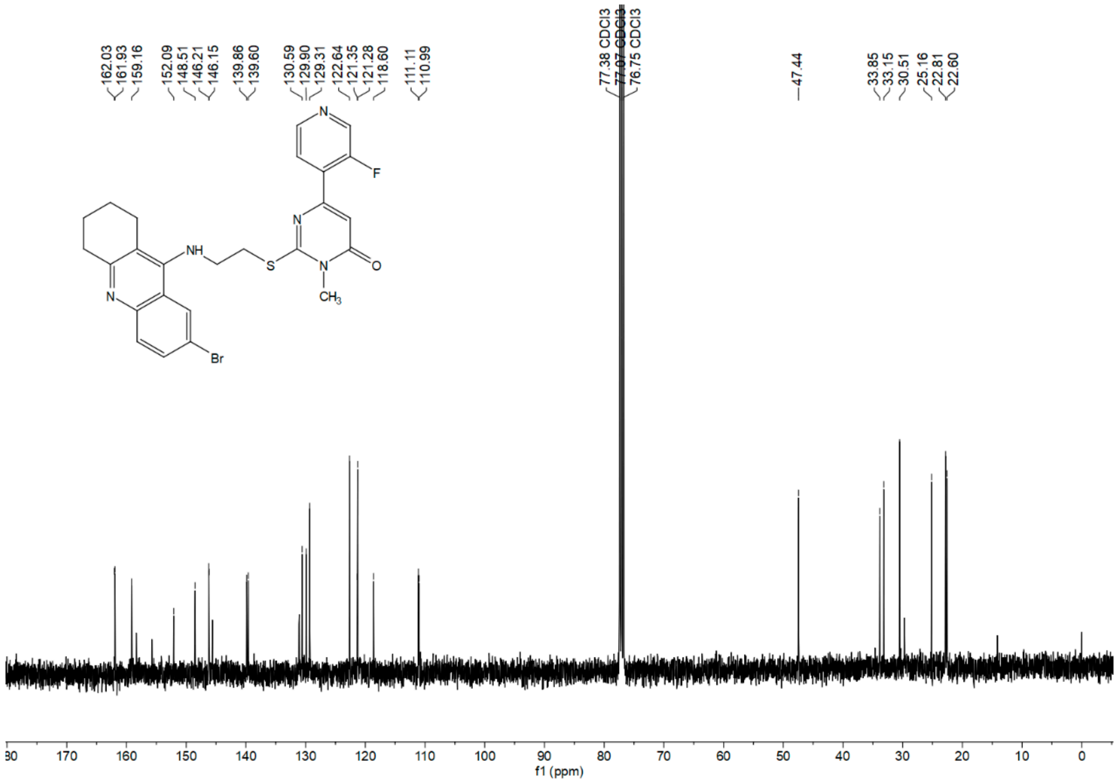

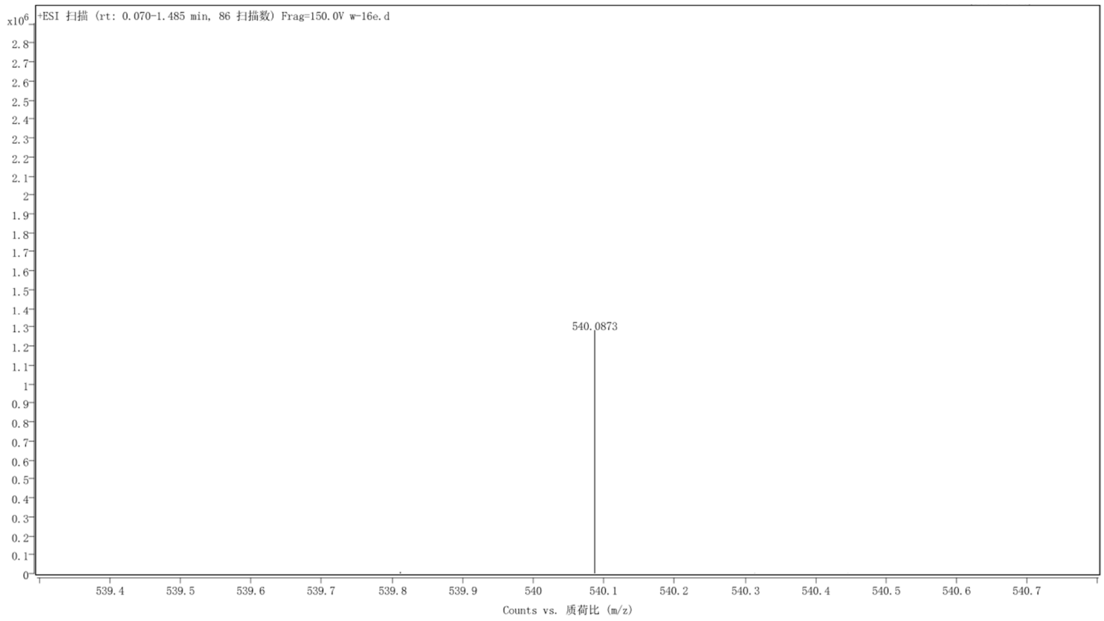

x10^6
+ESI 扫描 (rt: 0.070-1.485 min, 86 扫描数) Frag=150.0V w-16e.d
540.0873
Counts vs. 质荷比 (m/z)

NMR and HRMS of compound **16f**

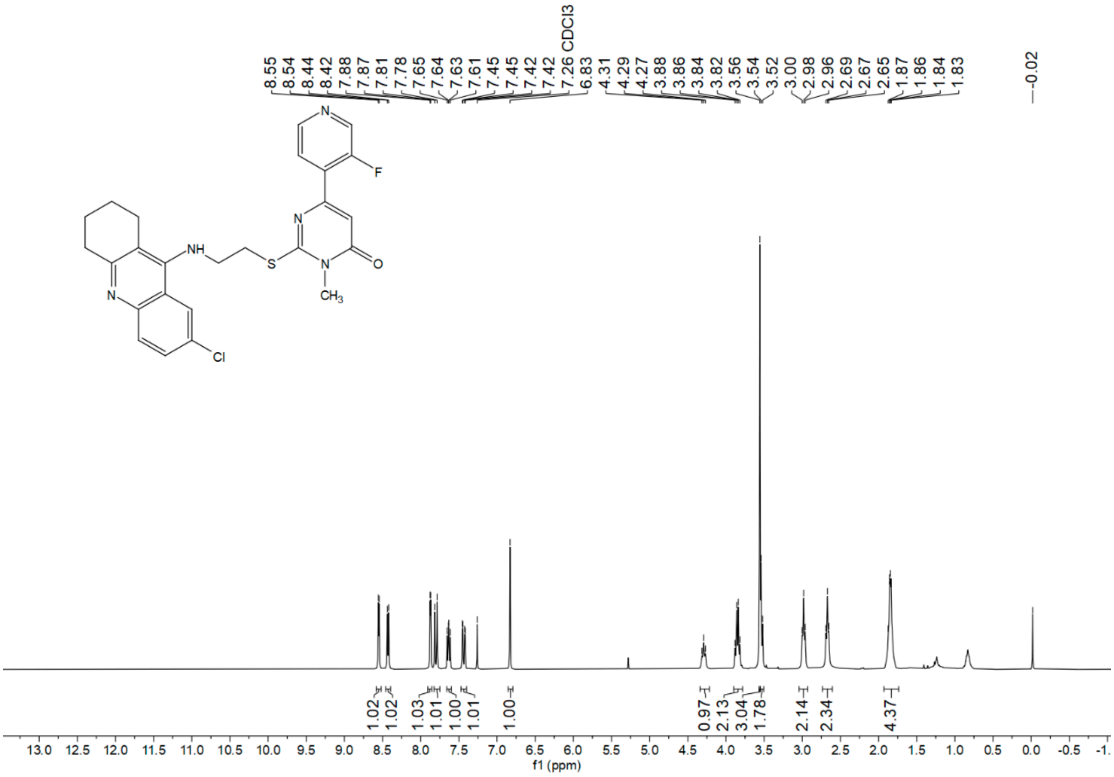

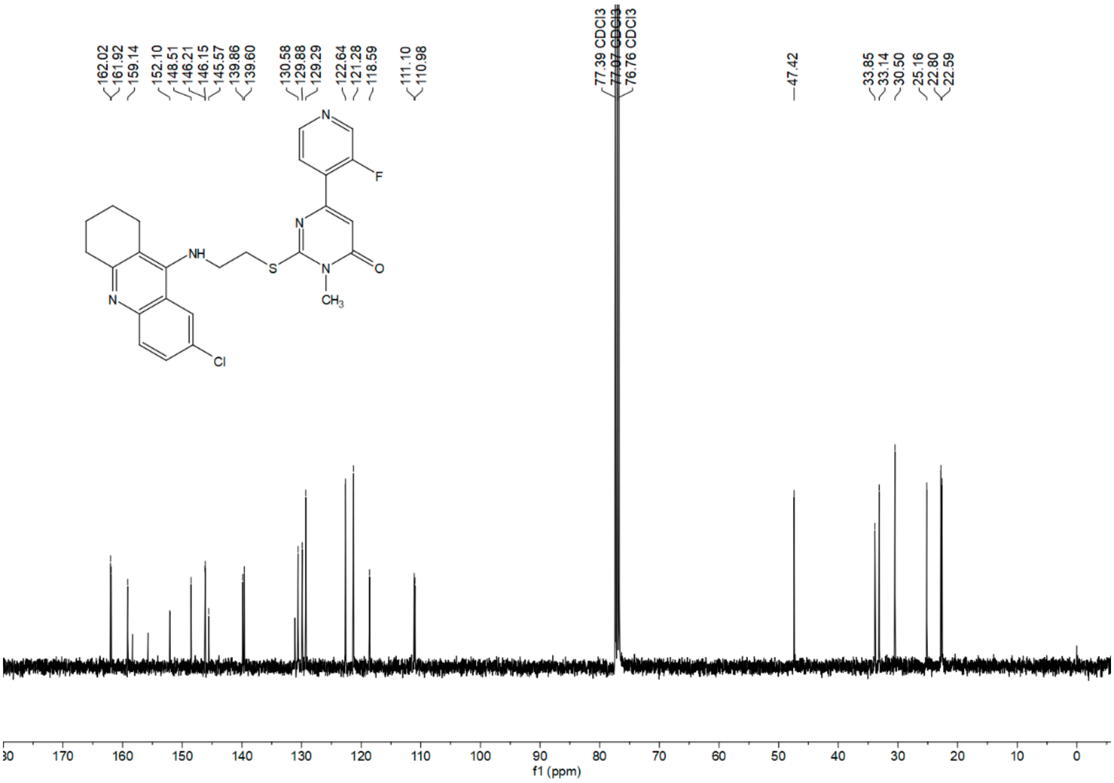

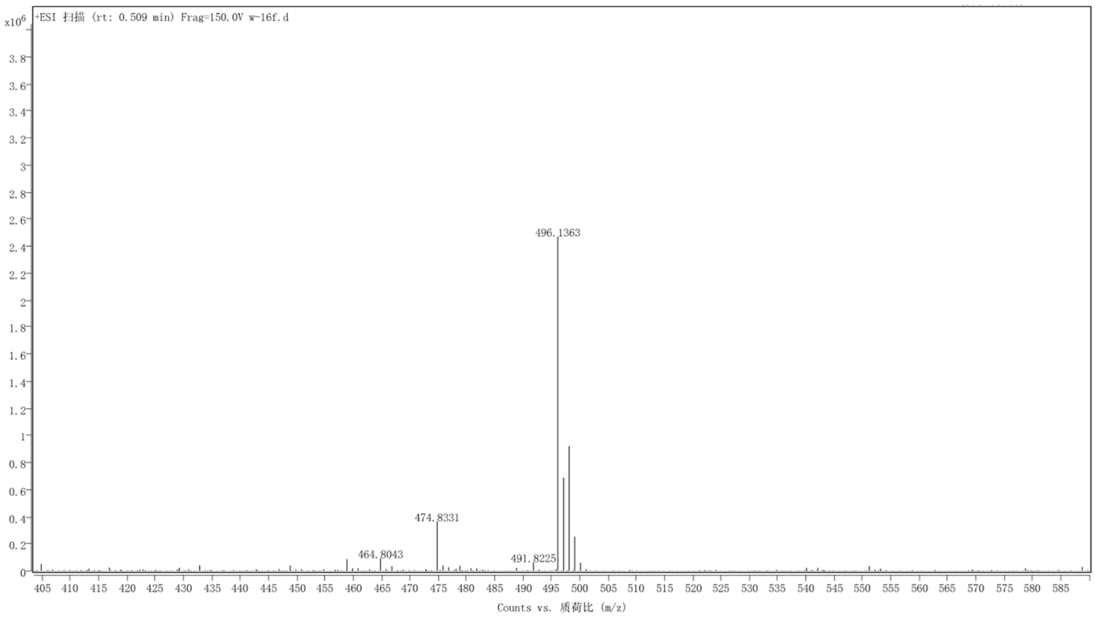
x10^6
+ESI 扫描 (rt: 0.509 min) Frag=150.0V w-16f.d
496.1363
474.8331
464.8043
491.8225
Counts vs. 质荷比 (m/z)

NMR and HRMS of compound **18a**

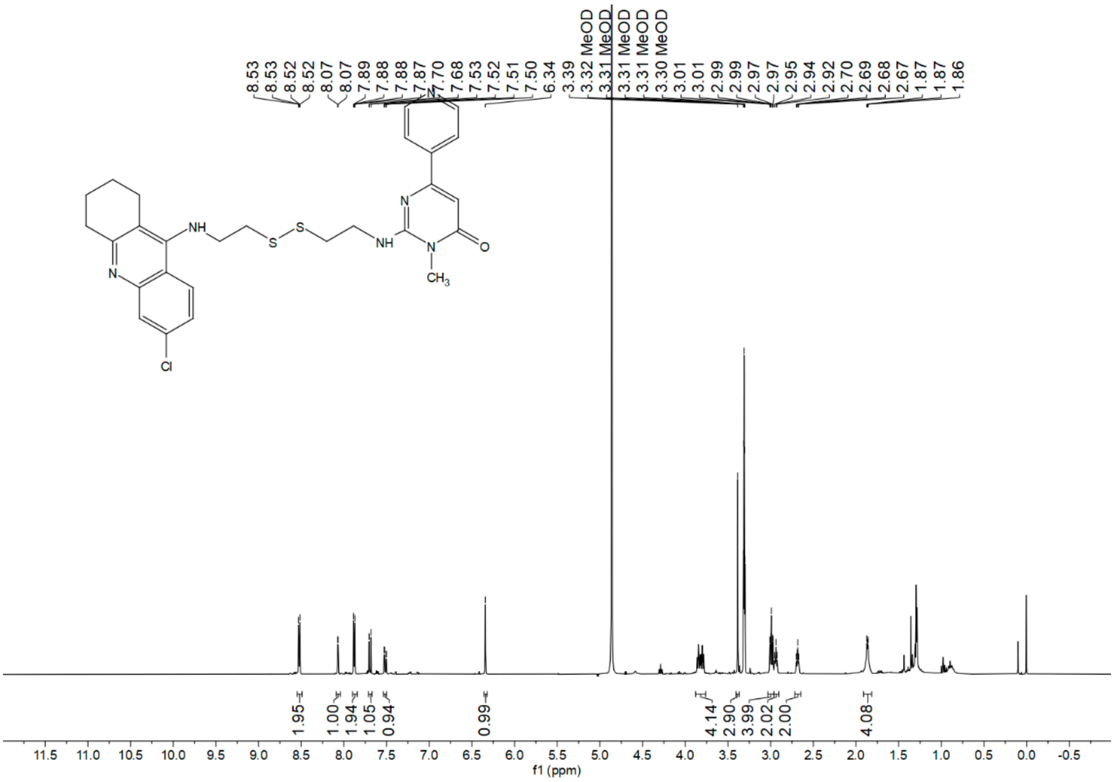

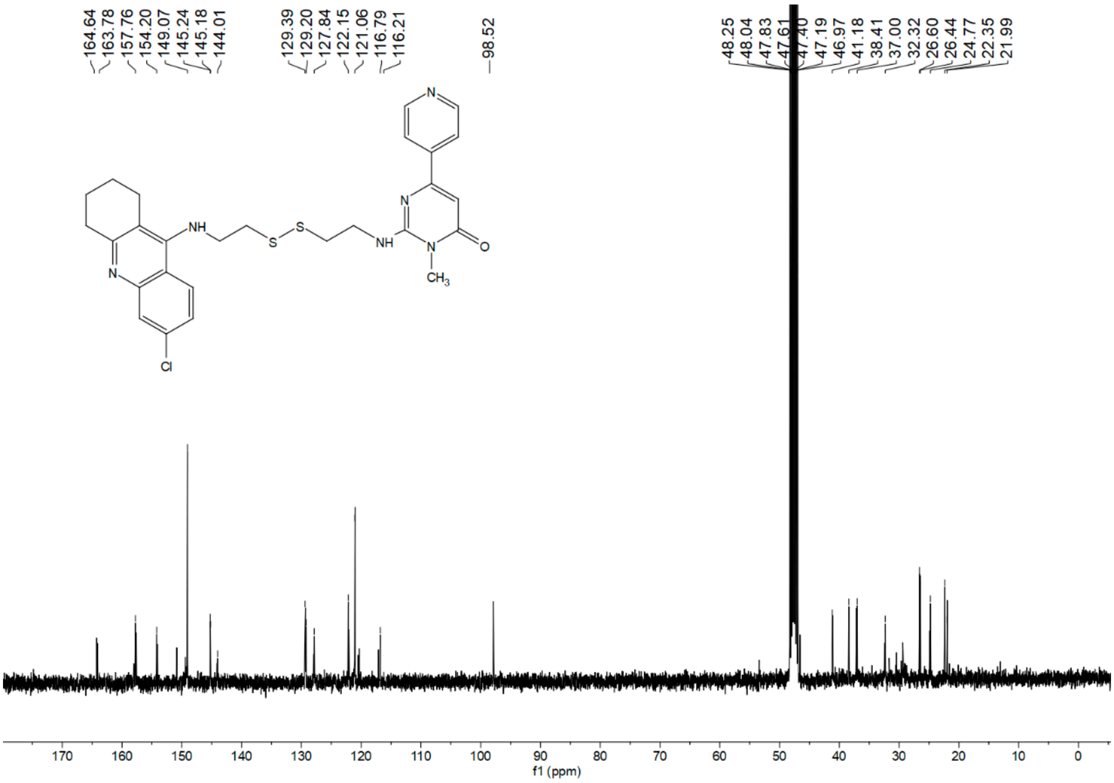

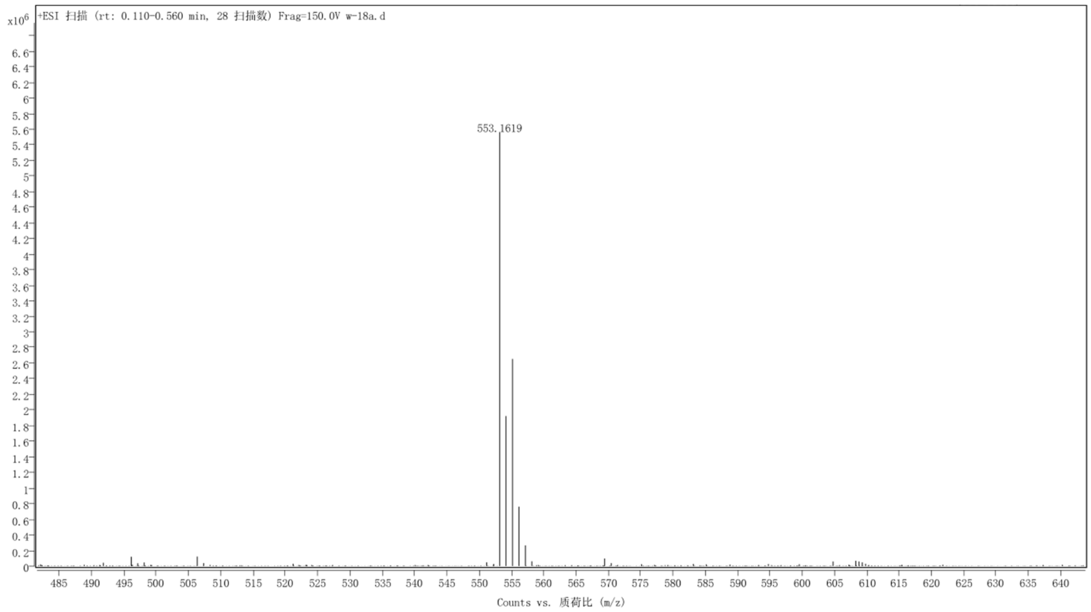

+ESI 扫描 (rt: 0.110-0.560 min, 28 扫描数) Frag=150.0V w-18a.d
x10^6
553.1619
Counts vs. 质荷比 (m/z)

NMR and HRMS of compound **18b**

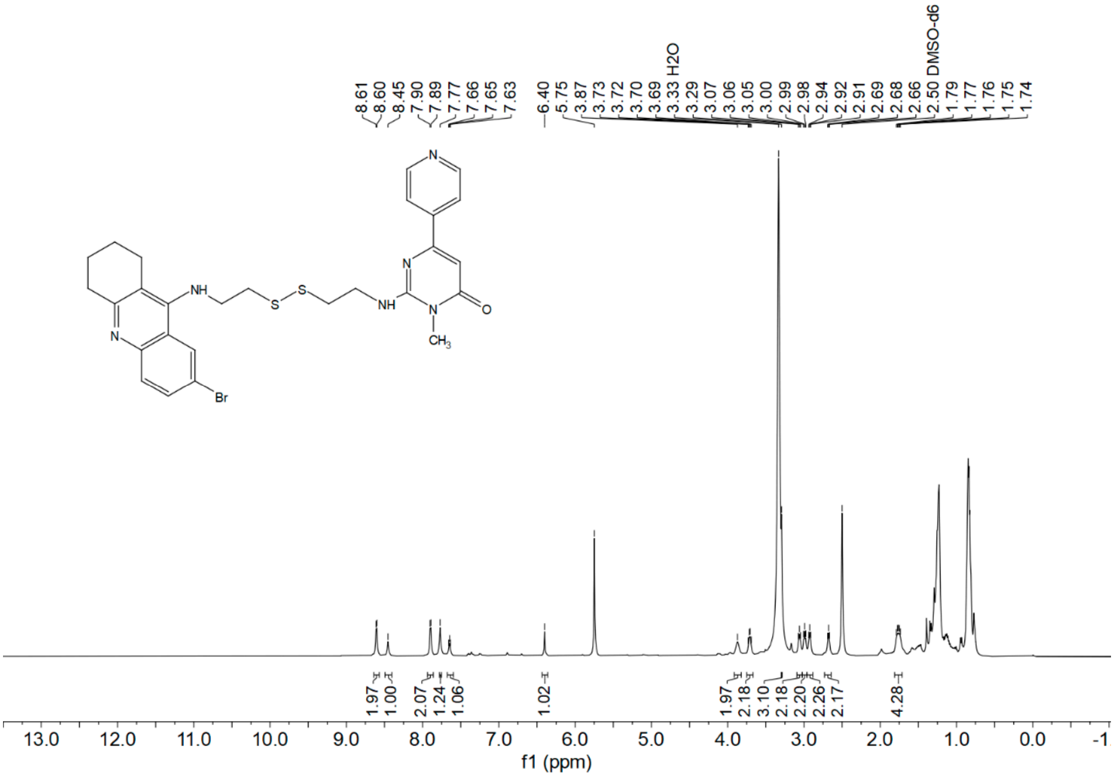

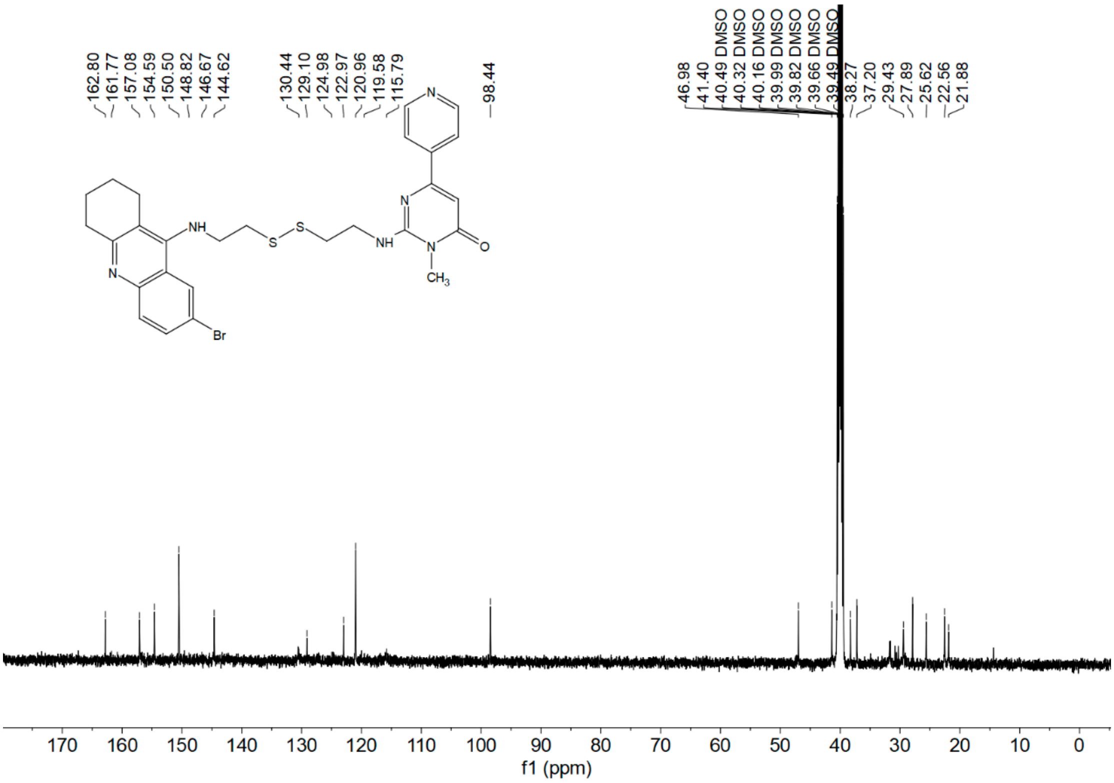

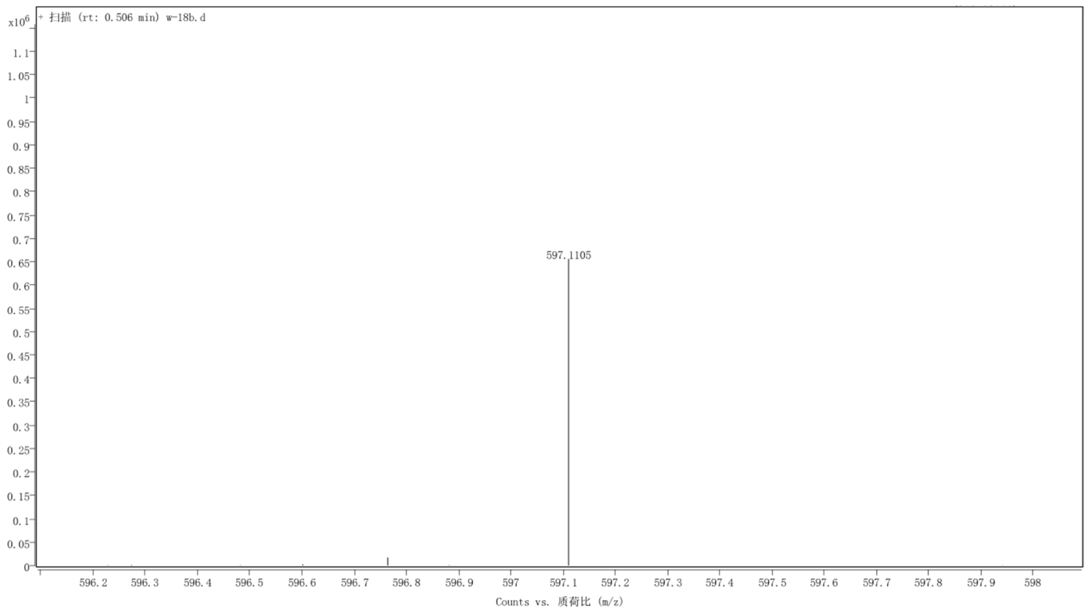

x10^6
+ 扫描 (rt: 0.506 min) w-18b.d
597.1105
Counts vs. 质荷比 (m/z)

NMR and HRMS of compound **18c**

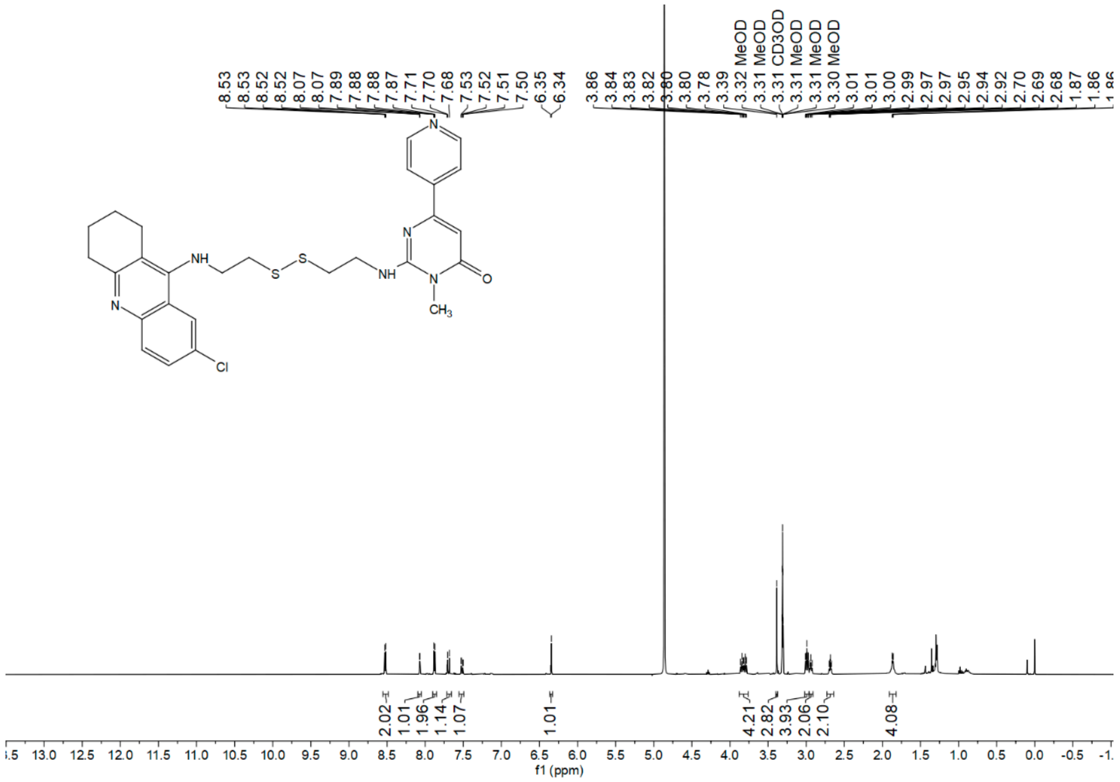

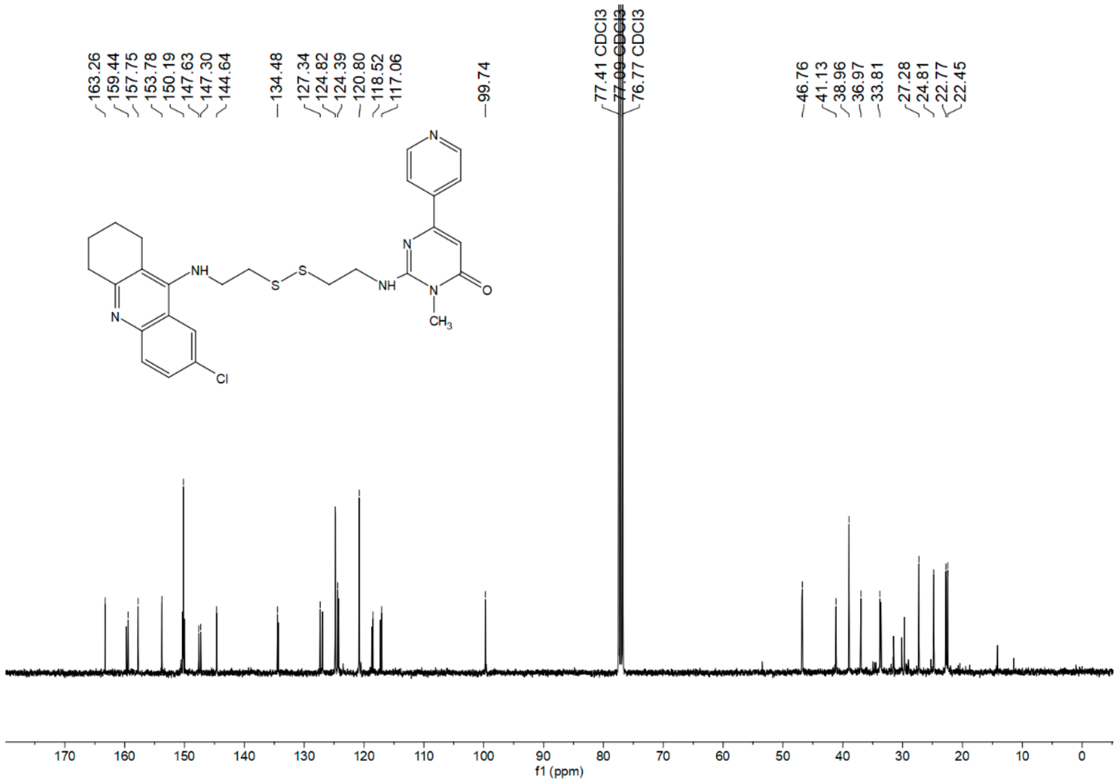

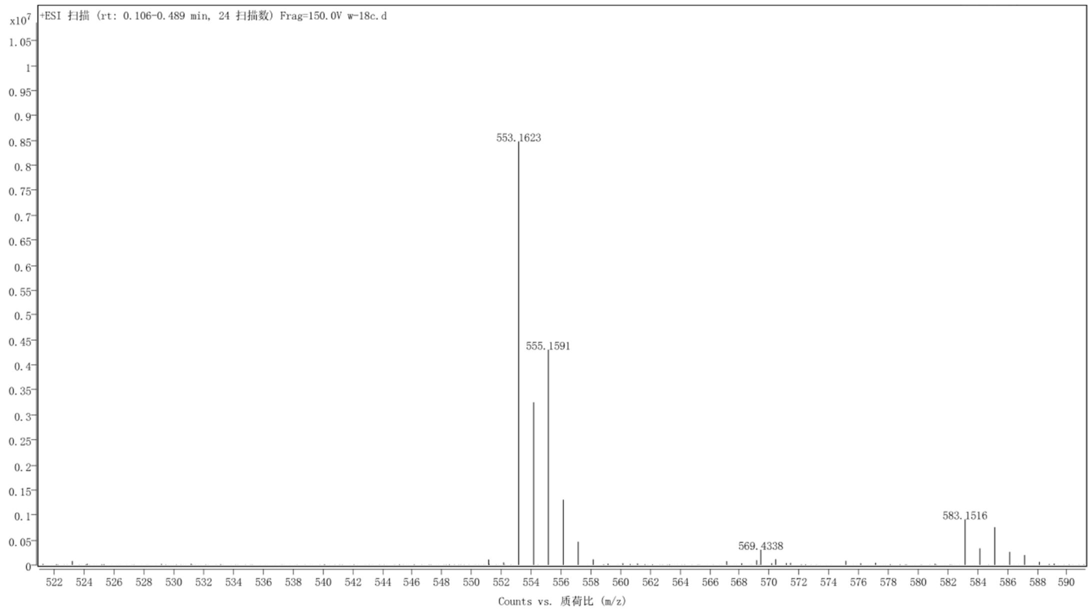
x10^7 +ESI 扫描 (rt: 0.106-0.489 min, 24 扫描数) Frag=150.0V w-18c.d
553.1623
555.1591
569.4338
583.1516
Counts vs. 质荷比 (m/z)
